# A chromosome-scale genome sequence of pitaya (*Hylocereus undatus*) provides novel insights into the genome evolution and regulation of betalain biosynthesis

**DOI:** 10.1038/s41438-021-00612-0

**Published:** 2021-07-06

**Authors:** Jian-ye Chen, Fang-fang Xie, Yan-ze Cui, Can-bin Chen, Wang-jin Lu, Xiao-di Hu, Qing-zhu Hua, Jing Zhao, Zhi-jiang Wu, Dan Gao, Zhi-ke Zhang, Wen-kai Jiang, Qing-ming Sun, Gui-bing Hu, Yong-hua Qin

**Affiliations:** 1 State Key Laboratory for Conservation and Utilization of Subtropical Agrobioresources/Guangdong Provincial Key Laboratory of Postharvest Science of Fruits and Vegetables/Key Laboratory of Biology and Genetic Improvement of Horticultural Crops (South China), Ministry of Agriculture and Rural Affairs/Lingnan Guangdong Laboratory of Modern Agriculture, College of Horticulture, South China Agricultural University, 510642 Guangzhou Guangdong, China; 2 Novogene Bioinformatics Institute, 100083 Beijing, China; 3 Horticulture Research Institute, Guangxi Academy of Agricultural Sciences, 530007 Nanning, Guangxi China; 4 Institute of Fruit Tree Research, Guangdong Academy of Agricultural Sciences/Key Laboratory of South Subtropical Fruit Biology and Genetic Resource Utilization (MOA)/Guangdong Province Key Laboratory of Tropical and Subtropical Fruit Tree Research, 510640 Guangzhou, China

**Keywords:** Plant evolution, Genome duplication

## Abstract

Pitaya (*Hylocereus*) is the most economically important fleshy-fruited tree of the Cactaceae family that is grown worldwide, and it has attracted significant attention because of its betalain-abundant fruits. Nonetheless, the lack of a pitaya reference genome significantly hinders studies focused on its evolution, as well as the potential for genetic improvement of this crop. Herein, we employed various sequencing approaches, namely, PacBio-SMRT, Illumina HiSeq paired-end, 10× Genomics, and Hi-C (high-throughput chromosome conformation capture) to provide a chromosome-level genomic assembly of ‘GHB’ pitaya (*H. undatus*, 2n = 2x = 22 chromosomes). The size of the assembled pitaya genome was 1.41 Gb, with a scaffold N50 of ~127.15 Mb. In total, 27,753 protein-coding genes and 896.31 Mb of repetitive sequences in the *H. undatus* genome were annotated. Pitaya has undergone a WGT (whole-genome triplication), and a recent WGD (whole-genome duplication) occurred after the gamma event, which is common to the other species in Cactaceae. A total of 29,328 intact LTR-RTs (~696.45 Mb) were obtained in *H. undatus*, of which two significantly expanded lineages, Ty1/copia and Ty3/gypsy, were the main drivers of the expanded genome. A high-density genetic map of F1 hybrid populations of ‘GHB’ × ‘Dahong’ pitayas (*H. monacanthus*) and their parents were constructed, and a total of 20,872 bin markers were identified (56,380 SNPs) for 11 linkage groups. More importantly, through transcriptomic and WGCNA (weighted gene coexpression network analysis), a global view of the gene regulatory network, including structural genes and the transcription factors involved in pitaya fruit betalain biosynthesis, was presented. Our data present a valuable resource for facilitating molecular breeding programs of pitaya and shed novel light on its genomic evolution, as well as the modulation of betalain biosynthesis in edible fruits.

## Introduction

Pitaya or pitahaya (*Hylocereus*), also referred to as dragon fruit, is one of the most important and widely spread fruit crops in tropical and subtropical countries. Pitaya likely originated from rainforests in tropical and subtropical areas of Latin America (Mexico and Colombia), and it is now a globally important fruit crop^1,2^. Due to its conspicuous appearance, shocking fuchsia colors, delicious taste, and high nutrients, as well as its antioxidant capacity and antiproliferative activities^3–5^, pitaya is becoming increasingly popular worldwide, with substantial increases in planted acreages and fruit production, according to the FAO (Food and Agriculture Organization) (http://faostat3.fao.org/).

Pitaya belongs to the *Hylocereus* genus in the family Cactaceae within the angiosperm order Caryophyllales. *Hylocereus* spp. are diploid (2n = 22), and 15 species of *Hylocereus* have been identified, among which only five are cultivated for producing fruits^6–8^. Currently, two types of pitaya fruit, namely, *H. undatus* (red peel with white pulp) and *H. monacanthus* (red peel with red pulp), are commercially produced at a large scale as fruit crops in Central America, Southeast Asia, and China^2^. A total of 12,946 scientific plant names at the rank of the species for the family Cactaceae have been recorded in the working list of plants (http://www.theplantlist.org/browse/A/Cactaceae/), among which 2047 are accepted species names. Among all the Cactaceae species, the columnar cacti (*Carnegiea gigantea*)^9^ and ‘David Bowie’ pitaya (*H. undatus*)^10^ genomes have been sequenced.

The potential nutrients and bioactive phytochemicals in pitaya fruit have been widely studied. In particular, pitaya is the only commercial edible fruit that contains high levels of betalains, which are water-soluble, tyrosine-originated alkaloid pigments restricted to the species of order Caryophyllales^11^. Their perspective beneficial properties in human health and nutrition are well documented, driven partly by their antioxidant effects^12,13^. Therefore, betalains in pitaya fruit are not only beneficial to human health but can also help consumers distinguish cultivars. In addition, betalains are considered a good resource for the food industry, as they are extensively utilized as economically important natural colorants of food and as functional foods^14^.

Betalains are categorized into two types, i.e., yellow betaxanthins and red betacyanins. The betalain biosynthetic pathway has been investigated in several Caryophyllales plants, such as beets, and microbial systems^15^. Briefly, betalains are synthesized via numerous enzymatic reaction steps, as well as spontaneous chemical reaction steps. To date, three core enzymes in the betalain biosynthesis cascade have been uncovered: ADH (arogenate dehydrogenase) catalyzes the formation of tyrosine^16^, DODA (4,5-DOPA extradiol dioxygenase) is responsible for the biosynthesis of betalamic acid^17^, and a cytochrome P450 enzyme referred to as CYP76AD1 catalyzes the synthesis of cyclo-DOPA^18^. In addition, in the synthesis of many plant secondary compounds, a basic skeleton is formed and modified with sugar moieties through the action of GTs (glycosyltransferase) and aromatic or aliphatic acyl moieties by ATs (acyltransferase)^19^. Betacyanins are formed by GTs and ATs, such as B6GT (betanidin 6-O-glucosyltransferase)^20^, B5GT (betanidin 5-O-glucosyltransferase)^21^, cDOPA5GT (cyclo-DOPA 5-O-glucosyltransferase)^22^, HCGT (hydroxycinnamate glucosyltransferase)^23^, and amaranthin synthetase^24^. Genes for these enzymes have been isolated in Caryophyllales species, and their expressions were observed to be upregulated alongside betalain accumulation. Moreover, transcription factors (TFs) have been reported to play critical roles in betalain biosynthesis. For instance, the anthocyanin MYB-like protein *BvMYB1* directly regulates the transcription of *CYP76AD1* and *DODA1*, thereby activating the betalain red pigment pathway^25^. Interestingly, BvMYB1 lost the bHLH-interacting residues and was unable to form the MBW (MYB-bHLH-WD40) complex, which provides a possible evolutionary mechanism reinforcing the mutual exclusion of betalains and anthocyanins^25^.

To date, several putative betalain biosynthetic genes, such as *TYR*, *GT-like, DOD-like*, and *CytP450-like*, have been discovered in pitayas based on transcriptomic analysis^26,27^. In addition, the WRKY TF HpWRKY44 was found to regulate the expression level of *CYP76AD1*^28^. However, compared to the precise regulatory networks of the anthocyanin biosynthesis pathway in fruits such as apples and pears, the modulation of betalain formation in pitaya fruit remains largely unknown. Furthermore, whole-genome resources for pitaya are not yet publicly available, which has greatly hindered the molecular breeding, biological research, and deep utilization of this increasingly popular fruit. Here, we sequenced and then assembled the chromosome-level genome of the *H. undatus* cultivar employing a combination of sequencing technologies, including PacBio Sequel SMRT Sequencing, Illumina HiSeq paired-end, 10× Genomics, and Hi-C (high-throughput chromosome conformation capture) sequencing. Moreover, we characterized pitaya genome evolution and transcriptome dynamics at different fruit development stages of *H. undatus* and *H. monacanthus* to decipher the global architecture of gene modulatory networks underlying betalain biosynthesis. The genome and transcriptome analyses presented herein shed novel light on the genome evolution and regulation of betalain biosynthesis in edible fruits and provide valuable resources for research on *Hylocereus* biology and breeding.

## Materials and methods

### Plant materials, DNA isolation, and genome sequencing

‘Guanhuabai’ (GHB, *H. undatus*) pitaya, a cultivar that has a red peel with white pulp, was utilized for genome sequencing. The DNA secure Plant Kit (Tiangen Biotech, Beijing, China) was employed to extract the total genomic DNA from young fresh pitaya stems. The quality of the isolated DNA was checked and used for processing the genome libraries. For SMRT sequencing with PacBio Sequel, at least 10 μg of sheared DNA was required for processing a 20-kb insert size library. SMRTbell template processing steps included the concentration of DNA, damage repair, end repair, hairpin adapter ligation, and template purification, as described by the manufacturer’s manual. Then, the processed genome library was run on the PacBio Sequel System (Pacific Biosciences, Menlo Park, CA, USA) for sequencing. For Illumina sequencing, processing of the short-read genomic library was performed with a library construction kit (Illumina, San Diego, CA) as described by the manufacturer. Overall, 7 paired-end genome sequencing reads of 250 bp to 10 kb inserts were processed and loaded onto the Illumina HiSeq × 10 platforms for sequencing. For 10× genomic library construction and sequencing, 1 ng of DNA (50 kb) was employed as the template for the GEM reaction step in the PCR, with the introduction of 16-bp barcodes into droplets. Then, the DNA in the droplets was sheared after purification of the intermediate DNA library. Four libraries were loaded onto the Illumina HiSeq × 10 for sequencing. For Hi-C sequencing, young stems were fixed by 1% formaldehyde solution in MS buffer (10 mM potassium phosphate, pH 7.0, 50 mM NaCl, 0.1 M sucrose) at room temperature in a vacuum. After fixation, the Hi-C library underwent restriction enzyme digestion, DNA end-repair, DNA ligase, and DNA fragmentation. Hi-C libraries were controlled for quality, and sequencing was performed on an Illumina HiSeq X Ten sequencer.

### De novo genome assembly

Before the de novo assembly of the pitaya genome, the genome size was estimated using the *k*-mer distribution assessment (*k* = 17) using 77.87 Gb of high-quality paired-end reads from Illumina short reads. The FALCON pipeline (https://github.com/PacificBiosciences/FALCON/, version 0.2.2) was employed to de novo assemble the long reads generated from the SMRT sequencing. We discarded PacBio reads smaller than 1 kb, while reads with a size of more than 10 kb were employed as seeds for error correction and assembly with the FALCON assembler. After that, Quiver was employed to polish the p-contigs (primary contigs) through alignment of the SMRT reads. Finally, Pilon was employed to conduct the second error correction round using the short paired-end reads generated by the Illumina HiSeq sequencer. The assembled length was 1.41 Gb with a contig N50 size of 579.57 kb. For the scaffolding step, SSPACE (version 3.0) was first employed to generate scaffolds from the HiSeq data from all the mate-pair libraries: 2 kb, 5 kb, and 10 kb. Afterward, the barcoded sequencing reads were employed to build superscaffolds using FragScaff (version 1-1). BWA software (version 0.7.8) was employed to map the Hi-C high-quality data to the scaffold genome, and unique reads were extracted for constructing chromosome-level assembly using LACHESIS software (version 201701).

Different methods were subsequently employed to explore the quality of the pitaya genome assembly process. The CEGMA (Core Eukaryotic Gene Mapping Approach, version 2.5)^29^ along with BUSCO (Benchmarking Universal Single-Copy Orthologs, version 4.0.5, embryophyta_odb10, 1614)^30^ were applied to explore the completeness of the assembled genome. We also assessed the completeness of the process of genome assembly by mapping the Illumina HiSeq-generated paired-end reads to the assembled genome using BWA (version 0.7.8)^31^.

### Genome annotation

An integration of de novo-based and homology-based approaches was employed to search the TEs (transposable elements) for annotation of the repetitive sequences. In the de novo-based method, we employed the RepeatModeler modeling package to identify de novo family repeats (http://www.repeatmasker.org/RepeatModeler.html, version 1.0.5), and LTR_FINDER (http://tlife.fudan.edu.cn/ltr_finder/, version 1.05) and RepeatScout (http://www.repeatmasker.org/, version 1.0.5) to generate a de novo repeat library. In the homology-based methods, we employed RepeatMasker (http://www.repeatmasker.org, version 3.3.0) against the Repbase TE data resource (version 15.02) and the RepeatProteinMask data resource (http://www.repeatmasker.org/) against the TE protein data resource.

To predict genes, we combined transcriptome-based, homolog-based, and de novo-based approaches. Homolog proteins from plant genomes, including *Chenopodium quinoa*, *Coffea canephora*, *Boea hygrometrica*, *Populus trichocarpa*, *Ananas comosus*, *Oryza sativa*, *Solanum tuberosum*, *Vitis vinifera*, *Beta vulgaris*, *Spinacia oleracea*, *Arabidopsis thaliana*, and *Daucus carota*, were retrieved and aligned to the pitaya genome assembly using TBLASTN^32^, with an *E*-value cutoff of 1e^−5^. We employed Solar software to join the BLAST hits^33^. The GeneWise web resource (https://www.ebi.ac.uk/Tools/psa/genewise, version 2.2.0) was employed to determine the exact gene structure of the respective genomic regions on each BLAST hit (Homo-set). In the transcriptome-based prediction methods, TopHat mapper (http://ccb.jhu. edu/software/tophat/index.shtml, version 2.0.8) along with the Cufflinks assembler (http://cole-trapnell-lab.github.io/cufflinks/, version 2.1.1) was employed to map the RNA sequencing data to the assembled genome. Moreover, we utilized RNA-Seq data to generate numerous pseudo-ESTs. After that, we mapped these pseudo-ESTs to the assembled pitaya genome, and then the PASA pipeline (http://pasapipeline.github.io/, version 2.3.3) was employed to predict gene models. This gene set was labeled the PASA-T-set and was employed in training the ab initio gene prediction approaches. Five ab initio gene prediction methods, namely, SNAP (http://korflab.ucdavis.edu/software.html, version 11-29-2013), GlimmerHMM (http://ccb.jhu.edu/software/glimmerhmm/, version 3.0.1), Genscan (http://genes.mit.edu/GENSCAN.html, version 1.0), Geneid (http://genome.crg.es/software/geneid/, version 1.4), and Augustus (http://augustus.gobics.de/, version 5.5), were employed to determine the coding regions of the repeat-masked genome. EvidenceModeler (EVM) software (http://evidencemodeler. sourceforge.net/, version 1.1.1) was employed to combine the gene model evidence from ab initio programs, Homo-set, PASA-T-set, and Cufflinks-set to create a non-redundant set of gene structures. A BLASTP search with an *E*-value of 1e^−5^ was performed against two integrated protein sequence data resources consisting of SwissProt (http://web.expasy.org/docs/swiss-prot_guideline.html, version 05-24-2016) and NR, for functional annotation of the coding sequences^32^. InterProScan (version 4.8) and HMMER (http://www.hmmer.org/, version 3.1) were employed to search against InterPro (http://www.ebi.ac.uk/interpro/, version 32.0) and Pfam (http://pfam.xfam.org/, version 27.0), respectively, for annotating the protein domains. The GO (Gene Ontology, http://www.geneontology.org/page/go, data resource) terms for each gene were acquired from the respective Pfam or InterPro entry. The KEGG (Kyoto Encyclopedia of Genes and Genomes, http://www.kegg.jp/kegg/kegg1.html, release 53) data resource was used to systematically classify gene functions (*E*-value ≤ 1e^−5)^.

ncRNAs (non-coding RNAs) in the pitaya genome were also predicted. Annotations of the rRNAs (ribosomal RNAs) were performed on the basis of their homology level with the rRNAs of numerous species of higher plants (not shown) via BLASTN search with an *E*-value of 1e^−5^. tRNAs (transfer RNAs) were determined with tRNAscan-SE software (tRNAscan-SE, RRID:SCR 010835, version 1.4) using the default settings. The Rfam data resource (http://rfam.xfam.org/, version 11.0) was searched using INFERNAL software (Infernal, RRID:SCR 011809, version 1.1) to identify miRNA (microRNA) and snRNA (small nuclear RNA) fragments^34^.

### Analyses of gene families and phylogenetic evolution

In addition to pitaya, the protein sequences of ten other plant species, *Rhodiola crenulate, Kalanchoe fedtschenkoi, Solanum lycopersicum, Arabidopsis thaliana, Beta vulgaris, Ananas comosus, Ipomoea nil, Phalaenopsis equestris, Spinacia oleracea,* and *Dianthus caryophyllus*, were downloaded from the genome database^35,36^. Then, all the genes of the 11 species were filtered as follows: (a) whenever there were many transcripts for one gene, we selected only the longest transcript of the coding region for downstream analysis, and (b) the genes coding for proteins not more than 30 amino acids long were filtered out. Next, we obtained the similarity relationship between all protein sequences for each species using BLASTP with an *E*-value of 1e^−5^. Paralogous and orthologous genes in these 11 species were clustered into gene families using the OrthoMCL program (http://orthomcl.org/orthomcl/, version 1.008) with an inflation setting of 1.5. In the phylogenetic analyses, we employed MUSCLE (version 3.7) to align the protein sequences of 419 single-copy gene families^37^ and then concatenated the alignments for each family into a super alignment matrix. Then, the maximum likelihood approach with 1000 bootstraps was employed to generate a phylogenetic tree of the 11 species in RAxML (http://sco.h-its.org/exelixis/web/software/raxml/index.html, version 8.0.0).

The MCMCtree program (http://abacus.gene.ucl.ac.uk/software/paml.html, version 4.5) implemented in the PAML package was employed to determine the divergence time on the basis of the phylogenetic tree. The MCMCtree running parameters included burn-in: 10,000, sample number: 100,000, and sample frequency: 2. The calibration times of divergence between *Ananas comosus* and *Phalaenopsis equestris* (95.0-124.0 Mya) and between *Solanum lycopersicum* and *Arabidopsis thaliana* (107.0-125.0 Mya) were abstracted from the TimeTree data resource (http://www.timetree.org/).

The CAFÉ program was employed to compare the cluster size differences between the ancestors of each species for assessment of the contraction along with the expansion of pitaya gene families^38^. The gene family changes along each lineage in the phylogenetic tree were explored using a random birth and death model. We introduced a PGM (probabilistic graphical model) to compute the likelihood of transitions from parent to child nodes based on gene family size in the phylogeny. The conditional probabilities were employed as the test statistics to calculate the corresponding *p*-values in each lineage, with a *p*-value of 0.05 used to uncover families that remarkably expanded and contracted.

### Insertion time and phylogenetic analysis of LTR-RTs

De novo searches were conducted for intact LTR-RTs (long terminal repeat retrotransposons) against the genome sequences using LTR_FINDER^39^ and LTRharvest^40^. The two ends of these LTR-RTs were aligned with Muscle (version 3.8.31)^37^, and the nucleotide divergence rate (λ) between the two LTR-RTs was filtered at a rate of more than 0.75. The genetic distance (K) was computed using the formula *K* = −0.75ln (1-4λ/3). The insertion time (T) of an LTR-RT was calculated with the formula *T* = K/2r, where r designates the nucleotide substitution rate, which was set at 7.0 × 10^−9^ substitutions/site/year^41^. All intact LTR-RTs with at least one protein domain were classified into Ty1/copia, Ty3/gypsy, and other superfamilies according to their structures and protein domains, which were identified based on the GyDB (*Gypsy* Database) by LTRdigest^42^. Nucleotide sequences of RTs were extracted from intact LTR-RT elements. Multiple alignments of the amino acid sequences of RTs without premature termination codons were performed using Muscle (version 3.8.31)^37^. NJ (Neighbor-joining) phylogenetic analyses were used to generate unrooted trees that were derived from uncorrected pairwise distances using TreeBeST (version 1.9.2) with the default parameters^43^. The classification of LTR-RTs into distinct lineages and clades was performed according to phylogenetic analyses^44^.

### Chromosome rearrangement analyses

Chromosome rearrangements in *H. undatus* were investigated using AEK (ancestral eudicot karyotype) genes^45,46^. The software MCscanX was employed to identify syntenic blocks shared between the AEK and the seven species (*V. vinifera*, *A. thaliana*, *M. domestica*, *F. tataricum*, *S. oleracea*, *B. vulgaris*, and *H. undatus*). Syntenic blocks containing more than 5 gene pairs were used to reconstruct the genome structure of the 7 selected species.

### Analyses of WGD events

BLASTP (*E*-value < 1e^−5^) was applied to perform a homolog search with the *H. undatus* genome, and MCScanX was employed to screen syntenic blocks. After that, Ks (synonymous substitution) values of paralogous blocks in *H. undatus* were computed using the FASTK pipeline (https://github.com/mrmckain/FASTKs), and the distribution of Ks values was employed to uncover putative WGD (whole genome duplication) events in *H. undatus*.

### Genetic map construction

A total of 203 F1 hybrid populations of ‘GHB’ × ‘Dahong’ (*H. monacanthus*, red peel with red pulp) and their parents were used to construct the genetic map. Raw reads were generated on an Illumina Hiseq^TM^ platform. Clean reads were processed from raw reads by removing reads containing adapter, poly-N, and low-quality reads. Clean reads were further obtained with *Mse*I digestion to prepare GBS (genotyping-by-sequencing) libraries and then aligned to the pitaya reference genome using BWA software. The GATK (Genome Analysis Toolkit, version 3.7) (Cambridge, MA, USA) was used to identify candidate SNPs (single-nucleotide polymorphisms) among the parents and F1 hybrid populations. The genetic map was constructed using Joinmap (version 4.1) with the maximum-likelihood method. The Kosambi algorithm was used to sort the markers of each group and calculate genetic distances.

### Transcriptome sequencing and analyses

To characterize the dynamic changes in gene expression during betalain accumulation, pulps from the 17^th^, 23^rd^, 25^th^, and 32^nd^ DAAP (the day after artificial pollination) were collected from ‘GHB’ (red peel with white pulp, *H. undatus*) and ‘Guanhuahong’ (GHH, red peel with red pulp, *H. monacanthus*) pitayas for transcriptome sequencing with three biological replicates.

The RNA Prep Pure Plant Kit (TIANGEN, China) was employed to extract total RNA as described by the manufacturer. Library preparation and RNA sequencing (RNA-Seq) was carried out by Novogene Biotechnology Corporation (Beijing, China). M-MLV Reverse Transcriptase (RNase H-) was employed to synthesize the first-strand cDNA using random hexamer primers. Thereafter, second-strand cDNA was generated with DNA polymerase I and RNase H, which was used to degrade the RNA strand. The library fragments were purified using the AMPure XP system (Beckman Coulter, Beverly, MA, USA) to preferentially filter and select 250–300 bp cDNA fragments. The quality of the cDNA library was evaluated on the Agilent Bioanalyzer 2100 platform. Afterward, Phusion High-Fidelity DNA polymerase, Universal PCR primers, and an Index (×) primer were employed to perform PCR, and the PCR products were subsequently purified by the AMPure XP system. The quality of the purified PCR products was checked using the Agilent Bioanalyzer 2100 platform. Subsequently, the TruSeq PE Cluster Kit v3-cBot-HS (Illumina) was employed to cluster the index-coded samples on a cBot Cluster Generation System as described by the manufacturer. Finally, the processed library was loaded onto the Illumina HiSeq Platform with 125 bp/150 bp paired-end reads for sequencing.

Raw fastq data (raw reads) were first processed with in-house Perl scripts. The adapters, as well as reads containing poly-N were filtered out to generate high-quality clean data (clean reads) for downstream analyses. The datasets were functionally annotated in comparison with the pitaya genome. The FPKM of each gene was computed by HTSeq (version 0.6.1). Differential expression analyses were carried out using the DESeq R package^47^, and DEGs (differentially expressed genes) were defined with an adjusted *p*-value < 0.05. After that, GO and KEGG enrichment of DEGs was implemented by the GOseq package in R and KOBAS software, respectively. The WGCNA network was created with the WGCNA package in R (version 3.2.5)^48^. Cytoscape (version 3.8.2) was employed to visualize the networks^49^.

### Betalain measurement and gene expression analyses

Betalains were extracted according to our previously described method^26^ and measured using spectrophotometry (Infinite M200, Tecan Co.) at 478 nm for betaxanthins and 538 nm for betacyanins. Total RNA was isolated using the EASYspin Plus Complex Plant RNA Kit (RN53) (Aidlab Biotechnology, Beijing, China). Single-stranded cDNA was synthesized using the PrimeScript™ RT Reagent Kit with gDNA Eraser (TaKaRa, Shiga, Japan). Quantitative reverse transcription PCR (qRT-PCR) was performed with a CFX384 Real-Time System (C1000 Touch Thermal Cycler, USA) using Real Universal Color PreMix (SYBR Green) (TIANGEN, China) with specific primers (Supplementary Table 1). All determinations were performed with three biological repetitions.

## Results and discussion

### Genome sequencing and assembly

On the basis of *K*-mer distribution assessment (*K* = 17), the estimated genome size of *H. undatus* (2n = 2x = 22 chromosomes) was 1.58 Gb while assembled genome size was 1.41 Gb, with heterozygosity and repeat contents of 0.65% and 65.99%, respectively (Table 1; Supplementary Fig. 1; Supplementary Table 2). Four technologies, PacBio, Illumina, 10× Genomics, and Hi-C, were combined to sequence and assemble the *H. undatus* genome and yielded a high-quality chromosome-level reference genome. Overall, 178.91 Gb of PacBio long reads (~113.23× coverage of the genome), 423.83 Gb of Illumina clean reads (from ~26.28× to 49.63× coverage of the genome), 237.98 Gb of 10× Genomic raw reads (~150.62× genome coverage), and 176.81 Gb of Hi-C data (~111.90× genome coverage) were generated, resulting in approximately 532.09-fold coverage of the *H. undatus* genome (Supplementary Table 3). The final assembled sequence was 1386.95 Mb, consisting of 675 scaffolds with a scaffold N50 of 127.15 Mb (Table 1) and 7647 contigs with a contig N50 of 0.58 Mb (Supplementary Table 4). The longest contig was 7.50 Mb and the longest scaffold was 146.67 Mb (Supplementary Table 4). The Hi-C approach was used to reorder and anchor the assembly sequence onto the 11 pseudochromosomes with lengths 97.47 to 146.67 Mb (Supplementary Fig. 2; Supplementary Table 5). The overall length of pseudochromosomes was responsible for 97.67% of the genome sequences, with a scaffold N50 number of 6 (Table 1; Supplementary Table 5). The genome of *H. undatus* had a GC content of 36.9% (Table 1; Supplementary Fig. 3).

**Table 1:** Assembly and annotation of the *H. undatus* genome

Estimated genome size (Gb)	1.58
Assembled genome size (Gb)	1.41
Number of scaffolds (≥2 kb)	675
Number of N50 scaffolds	6
N50 scaffold length (Mb)	127.15
Number of contigs (≥2 kb)	7,647
Number of N50 contigs	670
Longest chr (Mb)	146
GC content (%)	36.9
Transposable elements (%)	64.8
Predicted protein-coding genes	27,735
Average gene length (bp)	5,045.63
Average exon length (bp)	229.22

**Fig. 1:**
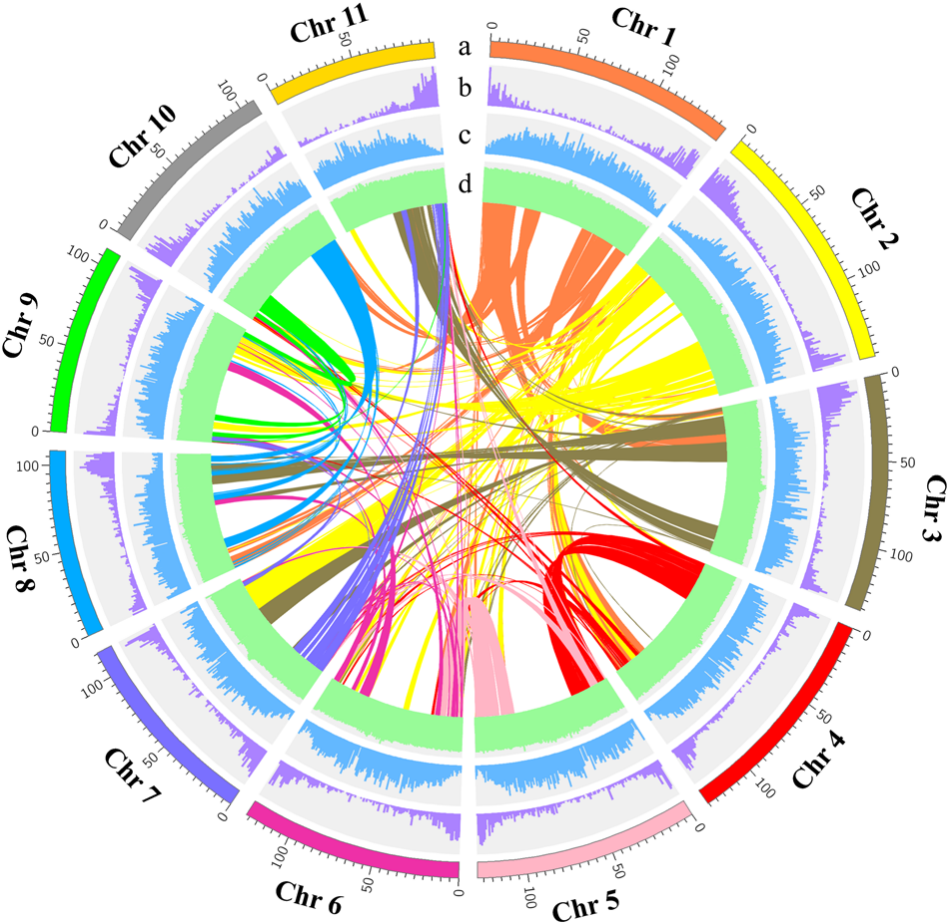
Landscape of *H. undatus* genome. Track a, the eleven chromosomes (in Mb scale). The density of genes (Track b) and TEs (Track c) across chromosomes. Track d, the GC contents. Colored lines in the center represent syntenic links between the chromosomes

To evaluate the quality of the genome assembly, BUSCO and CEGMA analyses were carried out. Consequently, we identified 93.8% and 93.55% completely conserved eukaryotic genes in the pitaya genome (Supplementary Tables 6–7), indicating a high completeness degree of the final assembly. BWA^31^ was also employed to explore the completeness of the genome assembly, which exhibited excellent completeness with mapping and coverage rates of 99.68% and 95.20%, respectively (Supplementary Table 8). In addition, the proportion of EST sequences with lengths longer than 500 bp, 1 kb, or 2 kb that could be mapped to the genome in one scaffold was more than 90% (Supplementary Table 9). All these results suggest a high degree of contiguity, as well as the completeness of the pitaya genome at the chromosome scale. Moreover, a total of 293 gene syntenic blocks along with 8450 paralogous gene groups were uncovered on the basis of the self-alignment of the 27,490 chromosome-anchored genes, revealing that the pitaya genome has undergone frequent segmental duplications and interchromosome fusions in its evolutionary history (Fig. 1).

### Genome annotation

A combined approach involving de novo predictions, RNA sequencing, and homology-based searches was employed to predict the genes encoding proteins in the *H. undatus* genome. Overall, 27,753 genes encoding proteins were predicted and annotated, with an average gene length of 5.05 kb and an average CDS length of 1159.29 bp (Table 1; Supplementary Table 10; Supplementary Fig. 4). A total of 95.1% (26,371 genes) of these genes could be annotated by homology to known proteins, domains, or expressed transcripts (Supplementary Table 11). Specifically, a total of 2,156 transcription factors (TFs) (7.78%) were uncovered in the *H. undatus* genome, and they were classified into 80 TF families consisting of 185, 168, and 80 members of MYB, bHLH, and WRKY, respectively (Supplementary Table 12).

On the basis of integrated analyses involving homology-based and de novo approaches, the *H. undatus* genome contained 896.31 Mb repetitive sequences (65.99% genome size) (Supplementary Table 13). Tandem duplications (microsatellites and small satellites) and interspersed repeats accounted for 0.56% of the genome (Supplementary Table 14). LTRs of retrotransposons accounted for the most abundant interspersed repeats (48.83% of the genome), followed by DNA transposable elements at 8.32% (Supplementary Table 14). The non-LTR retrotransposons LINEs (long interspersed nuclear elements) together with potential SINEs (short interspersed nuclear elements) occupied 5.25% of the sequenced genome (Supplementary Table 14). The genes annotated as ncRNAs included 4,989 miRNAs, 4,857 tRNAs, 5,909 rRNAs, and 3,877 snRNAs (Supplementary Table 15).

### Genome evolution and comparative genomic analysis

We retrieved the genome sequences of representative plant species and carried out comparative genomic analysis with *H. undatus* to infer the genome evolution and divergence time of *H. undatus*. The *Spinacia oleracea* and *Beta vulgaris* genomes had no additional genome duplication after the ancestral gamma hexaploidization, and *S. oleracea* could serve as a useful reference for exploring ancestral eudicot genome duplication events. The syntenic depth analysis indicated that there were multiple *H*. *undatus* blocks covering each *S. oleracea* gene (Supplementary Fig. 5A). Specifically, 75% of the *S. oleracea* genome had one or two syntenic blocks in *H*. *undatus*. In contrast, after a depth of 2×, the syntenic depth suddenly declined (Supplementary Fig. 5A), suggesting that there were two *H*. *undatus* blocks in each *S. oleracea* genome region and thus providing strong evidence for a distinct WGD event in *H*. *undatus*. The microsynteny profile reflects a 1:2 gene copy ratio between the *S. oleracea* and *H*. *undatus* genomes. Similarly, two distinct peaks of 0.3 and 0.7 appeared in the distribution of the 4dTv (4-fold transversion substitution rate) values between the *H*. *undatus* gene pairs (Supplementary Fig. 5B), indicating that the common ancestor of *H. undatus*, *S. oleracea*, and *B. vulgaris* had a WGT event and that a recent WGD event occurred in pitaya (Supplementary Figs. 5–6).

**Fig. 2:**
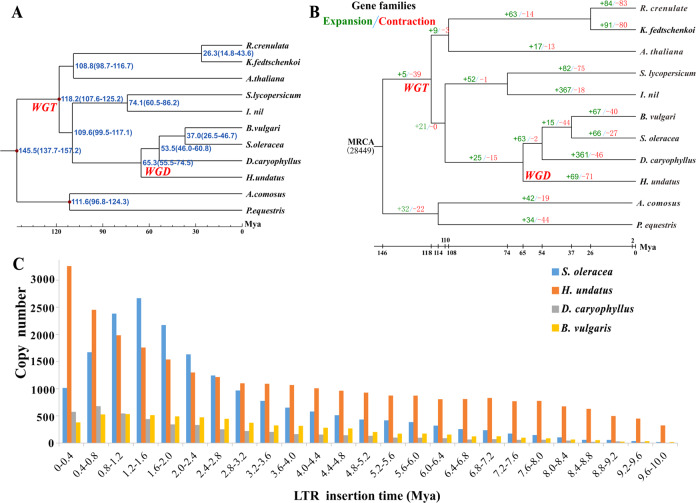
Species trees and LTR insertion times of *H. undatus*. **A** Species tree and divergence times of *H*. *undatus* and the other species. **B** Species tree and expansion-contraction in gene families. The green and red numbers present expanded and contracted gene families, respectively. MRCA stands for the most recent common ancestor. **C** Distribution of insertion times for LTR-RTs

A gene family cluster evaluation of the complete gene sets of Caryophyllales species (*H*. *undatus*, *B. vulgaris*, *S. oleracea* and *D. caryophyllus*) was performed. In total, 9,439 gene families were common in the four Caryophyllales species, and 871 gene families were unique to *H*. *undatus* (Supplementary Fig. 7A). The common gene families between the genes shared by *H*. *undatus* and 10 representative species (Supplementary Table 16) and pitaya plant-specific gene families were further investigated using the results from OrthoMCL data with an inflation parameter of 1.5. In total, 22,948 genes in 13,594 gene families were identified from the genome (Supplementary Fig. 7B; Supplementary Table 16). Of these, 419 single-copy orthologous gene families were common in the *H. undatus* genome and 10 other investigated plant species (Supplementary Fig. 7B). Subsequently, a phylogenetic tree was constructed to illustrate the divergence time and evolution of *H. undatus* with single-copy orthologous genes. The results showed that the divergence time between the *Hylocereus* and *Dianthus* species was estimated at ~65.3 (55.5–74.5) Mya (million years ago) based on the fossil-calibrated phylogeny (Fig. 2A). This result was consistent with a previous study in which Caryophyllales plants were separated before the split of asterids and rosids^50^. A total of 1,381 genes from 517 unique gene families were supported as pitaya-specific genes during its long evolutionary history (Supplementary Fig. 7B). Pitaya-specific genes were enriched in 45 GO terms consisting of 27 molecular function categories and 18 biological process categories, and most genes had terpene synthase activity (Supplementary Fig. 8A; Supplementary Table 17-1). According to KEGG analysis, most genes were enriched in plant-pathogen interactions, starch, and sucrose metabolism, and sesquiterpenoid and triterpenoid biosynthesis (Supplementary Fig. 8B; Supplementary Table 17-2).

Expanded and contracted gene families have been recognized as key drivers that shape the natural variation for adaptation in various species^51,52^. The CAFÉ program^38,53^ implemented with a PGM was employed to determine gene families that have undergone considerable expansion or contraction. This program regards the evolution of a gene family as a stochastic birth and death process in which the genes are gained and lost independently along each branch of the phylogenetic tree. Comparative genomic analyses were performed among *H. undatus* and ten representative plant species, and 69 (811 genes) and 71 (63 genes) gene family expansions and contractions were obtained, respectively, after divergence from *D. caryophyllus* (Fig. 2B), which suggests that more *H. undatus* gene families have experienced contraction relative to expansion during adaptive evolution. Expanded gene functions were enriched in 101 GO terms consisting of 40 molecular function categories (i.e., binding, lyase activity, terpene synthase activity) and 61 biological process categories (i.e., stress and defense response, organonitrogen compound biosynthetic process) (Supplementary Table 18). These results indicated that expanded genes are involved in defense and that stress compensation represents vital evolutionary targets^50^.

### Chromosome derivation analyses

Chromosome rearrangements in *H. undatus* were investigated using the approach describing ancestral eudicot karyotype (AEK) genes^45,46^. The total number of collinear genes was 11,342, accounting for 30.86% of the total gene sets of *H. undatus* and AEK, which was lower than the percentage in *V. vinifera* (48.19%), *M. domestica* (33.99%), and *A. thaliana* (31.19%) and higher than that of the other three species of Caryophyllales: *F. tataricum* (10.01%), *S. oleracea* (19.99%), and *B. vulgaris* (30.41%). The AEK gene composition of *H. undatus* and *F. tataricum* was much more complex than that of *S. oleracea* and *B. vulgaris* (Supplementary Fig. 9). In particular, both chromosomes 7 and 9 in *H. undatus* were composed of all seven inferred protochromosomes of AEK, and in *F. tataricum*, except for chromosome 5, all chromosomes were composed of seven protochromosomes. However, in *S. oleracea* and *B. vulgaris*, no chromosome was composed of all protochromosomes. Unlike *S. oleracea* and *B. vulgaris*, *H. undatus*, and *F. tataricum* both experienced WGD events, which may cause their chromosomal evolution to be more complicated. Chromosome 11 of *H. undatus* was relatively evolutionarily conserved and contained AEK genes from three ancient protochromosomes. On chromosome 11 of *H. undatus*, 85.82% of the total length of the collinearity block with the ancestor chromosome came from protochromosome 2. In total, predictions of a minimum of 80 chromosomal fissions, as well as 90 chromosomal fusions, occurred in *H. undatus*, resulting in its current structure of 11 chromosomes (Supplementary Fig. 9).

### LTR-RT expansion leads to large genome size in *H. undatus*

It has been suggested that plant genome size expansion is mainly affected by bursts of repetitive sequences such as LTR-RTs^54^. Thus, the evolution of LTR-RTs, as well as their possible contribution to the growth of the *H. undatus* genome, was investigated. *H. undatus* harbors the highest content of LTR-RTs (~696.45 Mb), in contrast with the other three closely related species of Caryophyllales: *B. vulgaris* (~122.67 Mb)^50^, *S. oleracea* (~470.12 Mb)^55^, and *D. caryophyllus* (~36.56 Mb)^56^. To trace the history of the expanded LTR-RTs in *H. undatus*, we identified LTR-RTs and estimated the insertion times of all intact LTR-RTs in these four species. A total of 29,328, 19,700, 6,891 and 5,333 intact LTR-RTs were identified in *H. undatus*, *S. oleracea*, *B. vulgaris*, and *D. caryophyllus*, respectively. In the genomes of *B. vulgaris* and *D. caryophyllus*, no significant proliferation of LTR-RTs was observed. In the genome of *S. oleracea*, LTR-RTs accumulated gradually in the last 8 Mya, and there was a burst in the recent 3 Mya, which peaked at approximately 1.2-1.6 Mya. Only in the genome of *H. undatus* were LTR-RTs continuously and substantially accumulated in the last 10 Mya, and the number of valid inserts increased over time, which showed a relatively longer expansion period compared with the other three species (Fig. 2C).

Approximately 68.50% of the intact LTR-RTs in the *H. undatus* genome had at least one protein-coding gene, of which the vast majority were Ty1/copia and Ty3/gypsy, accounting for 37.89% and 60.83%, respectively, and their corresponding total lengths were 64.74 Mb and 117.39 Mb. The proportion of Ty3/gypsy elements in the *H. undatus* genome was obviously higher than those in *S. oleracea* (43.86%), *B. vulgaris* (51.48%) and *D. caryophyllus* (54.60%) (Supplementary Table 19). Moreover, the total length of Ty3/gypsy elements in the *H. undatus* genome was 1.76, 5.55, and 9.35 times higher than that of *S. oleracea*, *B. vulgaris* and *D. caryophyllus*, respectively. Ty1/copia elements also expanded in *H. undatus*, with total lengths 6.82 and 3.52 times higher than those of *D. caryophyllus* and *B. vulgaris*, respectively (Supplementary Table 19). The evolutionary relationships of individual Ty1/copia and Ty3/gypsy LTR-RT superfamilies were analyzed in the four species. Ty1/copia elements in *S. oleracea*, *B. vulgaris* and *D. caryophyllus* were primarily separated into six evolutionary lineages, Angela, Ale, Bianca, Ivana, Maximus and TAR. However, Ty1/copia elements in *H. undatus* were assigned to five distinct lineages, of which the Bianca lineage was not found. Ty3/gypsy elements from these four species were all classified into six major evolutionary clades, Tekay, Galadriel, CRM, Reina, Athila and Tat. To understand the amplification of individual lineages, we calculated the copies in Ty1/copia and Ty3/gypsy. There was no significant expansion of Ty3/gypsy or Ty1/copia in *B. vulgaris* and *D. caryophyllus*, and the genomes of the two species were relatively small. In *S. oleracea*, Angela of Ty1/copia showed significant expansion, which accounted for 72.65% of the total expansion (Supplementary Fig. 10A; Supplementary Table 20). This may be one of the important reasons for the enlargement of the *S. oleracea* genome. Similarly, in *H. undatus*, the significantly expanded lineage of the Ty1/copia superfamily was Maximus, which accounted for 49.97% (Supplementary Fig. 10C; Supplementary Table 20), and the CRM of Ty3/gypsy also showed significant expansion, which accounted for 57.07% (Supplementary Fig. 10B; Supplementary Table 21). These results suggested that the two significantly expanded lineages may be drivers of the expanded genome of *H. undatus*.

### High-density genetic map of pitaya

A high-density genetic linkage map of pitaya was constructed by GBS technology using 203 F1 hybrid populations of ‘GHB’ × ‘Dahong’ and their parents. In total, 166.62 Gb, 1.91 Gb (18.13-fold tag coverage), and 1.94 Gb (16.3-fold tag coverage) of clean reads were generated from the 203 F1 populations, ‘GHB’ and ‘Dahong’, respectively (Supplementary Table 22-1). Subsequently, the clean reads were mapped to the pitaya genome, resulting in 98.75% average mapping rates and 16.13% average coverage rates of ‘GHB’ and ‘Dahong’ pitayas with a sequencing depth of 17.38× (Supplementary Table 22-2). In F1 populations, 99.17% average mapping rates and 9.75% average coverage rates were obtained with a sequencing depth of 11.5× (Supplementary Table 22-2). These results indicated that the reference pitaya genome had good coverage depth and coverage rates.

To identify high-quality SNP markers, reads with one locus in the pitaya genome were selected for SNP testing by the GATK UnifiedGenotyper tool. A total of 254,299 and 1,316,046 SNPs with 82.20% and 69.14% heterozygosis rates were obtained from ‘GHB’ and ‘Dahong’ pitayas, respectively (Supplementary Table 22-3), suggesting that both parents were equally heterozygous. In total, 793,759 SNP markers were identified and classified into eight segregation patterns (aa × bb, lm × ll, nn × np, ab × cc, hk × hk, cc × ab, ef × eg, ab × cd) (Supplementary Table 22-4). A total of 720,072 SNP markers could be used in the F1 populations based on the patterns lm × ll, nn × np, and hk × hk. A total of 56,380 SNP markers were obtained for linkage group analyses after removing the SNP markers with more than 10% missing data and filtering out distorted segregation with a *p*-value < 0.001.

A genetic map was constructed by Joinmap 4.1 software with the maximum-likelihood method. The filtered markers were clustered using various LOD (log-odds ratio) values (2 to 30). The Kosambi algorithm was used to sort the markers in each group and calculate the genetic distances. The genetic map of the female parent (‘GHB’) contained 4,979 bin markers spanning 2,710.78 cM, with an average intermarker distance of 0.54 cM and a maximum distance of 24.8 cM (Supplementary Fig. 11A; Supplementary Table 22-5). The genetic map of the male parent (‘Dahong’) contained 2,336 bin markers spanning 1,598.62 cM (centiMorgans), with an average intermarker distance of 0.68 cM and a maximum distance of 79.87 cM (Supplementary Fig. 11B; Supplementary Table 22-6). The integrated map contained 6,209 bin markers spanning 2226.22 cM, with an average intermarker distance of 0.36 cM and a maximum distance of 16.95 cM (Supplementary Fig. 12; Supplementary Table 22-7). These results suggested that a high-density genetic map was successfully constructed and that the high-resolution linkage map can be used for fine-mapping QTL (quantitative trait locus) in further research.

### Identification of betalain biosynthetic pathway genes

**Fig. 3:**
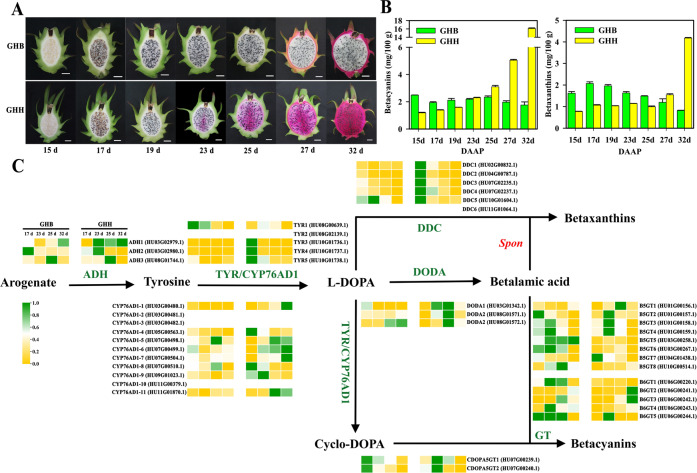
Genes involved in the betalain biosynthesis cascade. **A** The development of ‘Guanhuabai’ (GHB) and ‘Guanhuahong’ (GHH) pitaya pulp. **B** The betacyanin and betaxanthin contents of ‘GHB’ and ‘GHH’ pitaya pulp. **C** The expression profiles of genes related to betalain biosynthesis according to the RNA-Seq datasets of ‘GHB’ and ‘GHH’ pitaya pulp. The gene IDs are in brackets. *Spon*, spontaneous. Bar = 2 cm

Pitaya fruit is rich in nutrients and bioactive phytochemicals^2,57^. It is particularly noteworthy that pitaya is the only commercial edible fruit that accumulates high levels of betalains, including red betacyanins and yellow betaxanthins, during fruit maturation. In addition, the peel and pulp color is mainly determined by betalains. As shown in Fig. 3A, the pulp color of ‘GHH’ began to turn red at 23 DAAP and was fully red at 32 DAAP, while the pulp color of ‘GHB’ did not change during fruit maturation (Fig. 3A). Accordingly, in the ‘GHH’ pulp, the betacyanin and betaxanthin contents increased from 23 DAAP to 32 DAAP, from coloration to the mature stage, whereas their levels in the ‘GHB’ pulp remained relatively stable (Fig. 3B). Moreover, the contents of betacyanins, as well as betaxanthins, in the pulp of ‘GHH’ were remarkably higher than those in ‘GHB’ pulp during fruit maturation (Fig. 3B).

The structural genes *ADH*, *TYR* (*tyrosinase*), *DDC* (*tyrosine*/*DOPA decarboxylase*), *CYP76AD1*, *DODA,* and *GT* have been shown to be involved in betalain biosynthesis (Fig. 3C)^58–60^. In the *H. undatus* genome, *ADH* underwent a gene duplication event that gave rise to two clades, deregulated ADHα and canonical tyrosine-sensitive ADHβ. Three *ADH* genes were obtained from the pitaya genome, of which *ADH1* and *ADH3* were clustered into the ADHα clade, while *ADH2* belonged to ADHβ (Supplementary Fig. 13A). The *CYP76AD* lineage is closely related to the *CYP76T* and *CYP76C* families of *CYP P450* genes^18^. These genes underwent two gene duplications and gave rise to three paralogous lineages, namely, *CYP76ADα*, *CYP76ADβ*, and *CYP76ADγ*. Eleven genes were annotated as *CYP76AD1*, of which *CYP76AD1-1*, *CYP76AD1-2*, and *CYP76AD1-10* grouped in the CYP76AD1-α clade, *CYP76AD1-3* grouped in the CYP76AD1-β clade, and the other seven genes divided into the CYP76AD1-γ clade (Supplementary Fig. 13B). The *DODA* lineage falls into the *LigB* gene family and is widespread in land plants, from bryophytes to angiosperms^61^. The DODA lineage experienced gene duplication and resulted in two main clades, termed DODAα and DODAβ^62^. In addition, multiple further duplications have occurred within this clade. In the pitaya genome, *DODA1* clustered in the DODA1 clade, *DODA3* clustered in the DODA2 clade, and *DODA2* clustered in the LigB clade (Supplementary Fig. 13C). Betalain-related GT enzymes (B5GT, B6GT, and cDOPA5GT) arise from flavonoid-related GTs and are broadly conserved and distinct from each other across Caryophyllales. The results from the phylogenetic analysis suggested that two *cDOPA5GT* genes (*cDOPA5GT1* and *cDOPA5GT2*) were closer to *Mirabilis jalapa cDOPA5GT*, eight *B5GTs* (*B5GT1*, *B5GT2*, *B5GT3*, *B5GT4*, *B5GT5*, *B5GT6*, *B5GT7,* and *B5GT8*) grouped in the B5GT clade, and five *B6GTs* (*B6GT1*, *B6GT2*, *B6GT3*, *B6GT4,* and *B6GT5*) were closer to *Dorotheanthus bellidiformis* and *Cleretum bellidiforme B6GTs* (Supplementary Fig. 13D). In addition, three *TYRs* (*TYR1*, *TYR2*, and *TYR3*) and six *DDCs* (*DDC1*, *DDC2*, *DDC3*, *DDC4*, *DDC5,* and *DDC6*) were identified, which probably had catalytic activity for the formation of cyclo-DOPA and decarboxylated betalains, respectively. In total, 43 genes involved in betalain biosynthesis were identified in the *H. undatus* genome.

To further investigate the genetic information underlying betalain biosynthesis, we performed transcriptomic observations during pitaya fruit maturation based on the high-quality genome. Four key developmental stages (17, 23, 25, and 32 DAAP) of ‘GHB’ and ‘GHH’ pitaya pulp were sampled for transcriptome sequencing. Based on the heatmap of 43 betalain-related genes, *ADH1*, *CYP76AD1-1*, *CYP76AD1-6*, *CYP76AD1-7*, *CYP76AD1-11*, *DODA1*, *DODA2*, *B5GT1,* and *B6GT2* were specifically highly expressed during pulp maturation of ‘GHH’ pitaya, which was rich in betalains (Fig. 3C; Supplementary Table 23).

According to the transcriptome analysis, 31,355 genes were expressed at diverse developmental stages, and 3,768 DEGs changed over the course of fruit maturation with padj < 0.05. In detail, 824 (322 upregulated and 502 downregulated DEGs), 780 (405 upregulated and 375 downregulated), 1,191 (738 upregulated and 453 downregulated), and 973 DEGs (448 upregulated and 525 downregulated) were expressed at 17 d, 23 d, 25 d and 32 d between ‘GHB’ and ‘GHH’ pitaya pulp, respectively (Supplementary Fig. 14A; Supplementary Table 24). Thus, the pairwise comparison between ‘GHB’ and ‘GHH’ pitaya pulp at the same stages suggested that numerous genes were reported to be differentially expressed at 25 d and 32 d. From the Venn diagrams, three upregulated DEGs were expressed at 17 d, 23 d and 25 d, while 52 upregulated DEGs were expressed at 23 d, 25 d, and 32 d between ‘GHB’ and ‘GHH’ pitaya pulp (Supplementary Fig. 14B; Supplementary Table 25-1). Six downregulated DEGs were expressed at 17 d, 23 d, and 25 d, while 82 downregulated DEGs were expressed at 23 d, 25 d, and 32 d between ‘GHB’ and ‘GHH’ pitaya pulp (Supplementary Table 25-2). Interestingly, seven DEGs were expressed across all stages between ‘GHB’ and ‘GHH’ pitaya pulp. Except for three novel genes, four DEGs (*HU03G00203.1*, *HU03G00478.1*, *HU03G00480.1,* and *HU03G01342.1*) located on chromosome 3 and annotated as acetyltransferase, cytochrome c, CYP76AD1-1, and DODA1 were obtained (Supplementary Table 25-3). In addition, the DEGs in the white and red pitaya pulp based on the trinity database at the 23^rd^, 25^th^, and 32^nd^ DAAP were compared using BLAST to select the best and longest transcripts for Ka and Ks analyses. Forty-seven positive genes were considered to be candidate genes involved in betalain biosynthesis based on their selection during evolution (Supplementary Table 26). These genes clustered into 181 GO terms and 33 KEGG pathways (Supplementary Fig. 15A-B). Eight genes were highly expressed in 32 d ‘GHB’ pitaya pulp, and eleven genes were highly expressed in 25 d or 32 d ‘GHH’ pitaya pulp (Supplementary Fig. 15C).

**Fig. 4:**
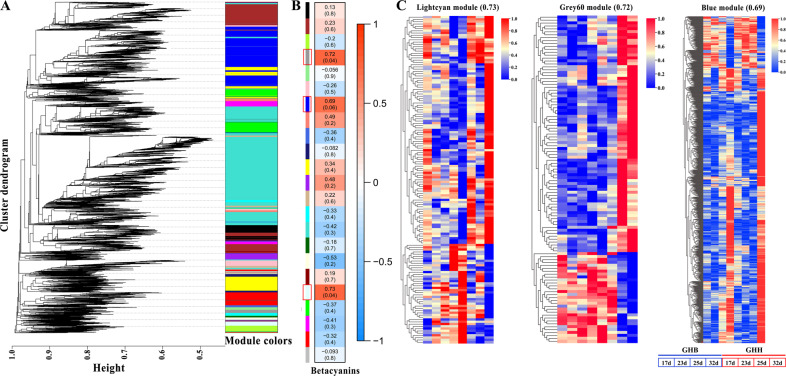
Transcriptional regulation of betalain biosynthetic genes. **A** WGCNA dendrogram indicating the expression of 23 different gene modules in all 8 pitaya samples. **B** Trait and module association analyses. Different colors designate the 23 different modules. **C** Heatmap of three highly correlated modules, including light cyan (145 genes), grey60 (112 genes), and blue (825 genes) modules

WGCNA was carried out to investigate the coexpression networks of DEGs in the transcriptome data, in which all the coexpressed genes were connected to each other with varying association strengths. As shown in Fig. 4A, genes were partitioned into 23 coexpression modules. Based on the correlation between gene modules and betacyanin contents, we established that three of the 23 coexpression modules were positively linked to betacyanin contents (Fig. 4B: lightcyan, 0.73; grey60, 0.72; and blue, 0.69). For further understanding of those three modules, the heatmap of light cyan (145 genes), grey60 (112 genes), and blue (825 genes) suggested that most genes were remarkably more highly expressed in ‘GHH’ pitaya than in ‘GHB’ pitaya (Fig. 4C; Supplementary Table 27). Thus, these results provide new directions and useful candidate genes for investigating the modulation of betalain biosynthesis.

### Identification of potential transcription factors involved in betalain biosynthesis

**Fig. 5:**
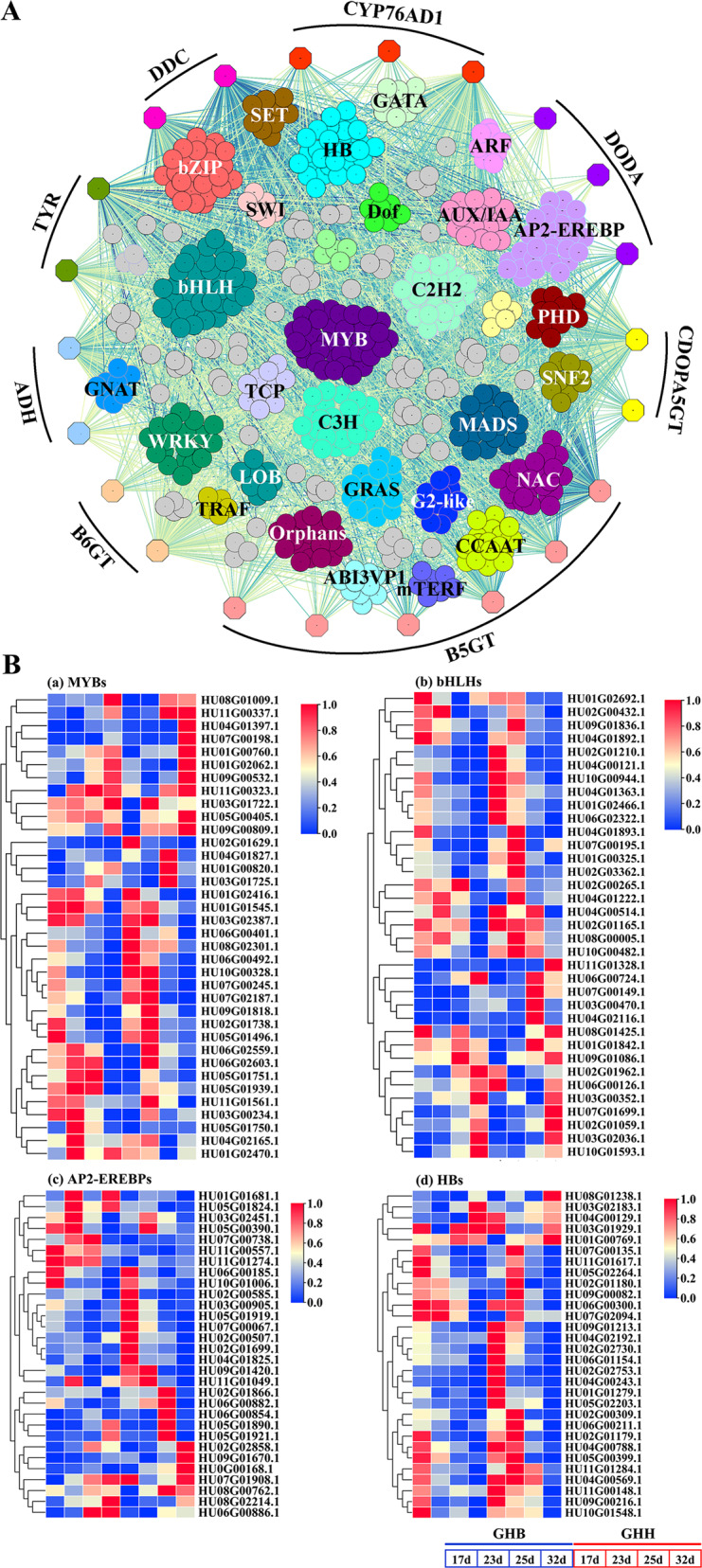
Transcriptional modulation of betalain biosynthetic genes. **A** The coexpression network connecting the structural genes in betalain biosynthesis with the transcription factors representing the modulation of betalain biosynthetic genes. Expression associations between TFs and catechin-associated genes (colored solid hexagons) are indicated with colored lines according to weight. **B** Heatmap of MYBs, bHLHs, AP2-EREBPs, and HBs, which had the maximum number in the coexpression network. Orange circles indicate candidate TFs highly expressed at 25 d and/or 32 d in ‘GHH’ pitaya

Accumulating evidence demonstrates that the production of plant secondary metabolites is likely to be modulated at the transcriptional level, which generally depends on the crosstalk of DNA-linked mechanisms and the activity of TFs that may act in an integrated approach. Transcriptional regulation of anthocyanin biosynthesis has been intensively investigated, and many TFs, including MYB, bHLH, AP2, and AP2/ERF, have been reported to play crucial roles in controlling anthocyanin biosynthesis^63,64^. Specifically, it is well recognized that the MBW transcriptional activation complex that is composed of R2R3-MYB, bHLH, and WD40 proteins acts as the major determinant regulator of anthocyanin biosynthesis by directly regulating the expression of anthocyanin structural genes^65^. Previously, only a few TFs, such as BvMYB1 and HpWRKY44, were found to regulate betalain biosynthesis in beet and pitaya, respectively^25,28^. Intriguingly, the MBW complex has been implicated in betalain biosynthesis^66^, but the regulatory mechanism remains largely unknown. Therefore, comprehensive and large-scale identification of potential TFs and their interactions with betalain biosynthetic genes are essential to gain insights into the molecular basis of betalain biosynthesis in fruits. The expression patterns of *ADH*, *TYR*, *DDC*, *CYP76AD1*, *DODA*, *CDOPA5GT*, *B5GT,* and *B6GT* were closely linked to the expression patterns of 557 TFs belonging to 66 families, mainly MYB, bHLH, AP2-EREBP, HB, NAC, Orphans, WRKY, and bZIP TFs, and other families that play a pivotal role in plant growth, development, and secondary metabolism (Fig. 5; Supplementary Fig. 16; Supplementary Table 28). Subsequently, the heatmap of the four largest number of TFs, including 36 MYBs, 35 bHLHs, 30 AP2-EREBPs, and 30 HBs, showed that two *MYBs* (HU04G01397.1, HU07G00198.1), four *bHLHs* (HU03G00470.1, HU04G02116.1, HU07G00149.1, HU11G01328.1), five *AP2-EREBPs* (HU02G01866.1, HU06G00882.1, HU06G00854.1, HU09G01670.1, HU0G00168.1) and an *HB* (HU08G01238.1) were correlated with *CYP76AD1-1* and *DODA1* expression patterns, which were highly expressed at 25 d and 32 d in the ‘GHH’ pitaya (Fig. 5B). Thus, these twelve TFs might be crucial transcriptional regulators of betalain biosynthesis in the pitaya fruit.

A potential mechanism underlying pitaya betalain biosynthesis at different levels in white and red pulp was proposed (Supplementary Fig. 17A). Except for *GTs* that were irregularly located on chromosomes 1, 3, 4, 6, 7, and 10 and without specific expression patterns in red and white pitaya pulp (Fig. 3C, Supplementary Table 23), the other three structural genes *ADH1*, *CYP76AD1-1,* and *DODA1* (the homologous genes of *ADHα*, *CYP76AD1α*, and *DODAα*, respectively) were all located on chromosome 3 with different transcriptional directions (Supplementary Fig. 17A). *ADH1*, *CYP76AD1-1,* and *DODA1* were highly expressed in the red pulp, resulting in more betalains being produced in red pulp than in white pulp (Supplementary Fig. 17B). These findings suggested that the clustering of *ADH1*, *CYP76AD1-1,* and *DODA1* located on chromosome 3 is likely responsible for the genetic stability of betalain heredity, and their differential expressions are accountable for different betalain biosynthesis contents in white and red pulp. The TFs and target genes identified in this work will help to elucidate the transcriptional regulatory network involved in betalain accumulation in pitaya.

## Conclusions

The high-quality, chromosome-level genome assembly of pitaya was obtained using PacBio and Illumina sequencing platforms and Hi-C technology. The assembly had a 1.41 Gb overall size, and the scaffold N50 reached 127.15 Mb. In total, 27,753 protein-coding genes were predicted, and 26,371 genes (95.1%) were annotated. Comparative genomic analysis revealed that a WGT event and a recent WGD event occurred in pitaya and that the recent event occurred after the gamma event. By GBS technology, a high-density genetic map was constructed for ‘GHB’ and ‘Dahong’ pitayas and their F1 populations. Remarkably, using detailed transcriptome data, we presented a global view of the regulatory network of betalain biosynthesis in pitaya fruit and provided many potential candidate genes to fully reveal the betalain biosynthetic cascade in pitaya. Given the economic importance of pitaya, the genomic data in this study offer valuable information for a better understanding of genome evolution in Caryophyllales and provide valuable gene resources for genetic improvement aimed at pitaya fruit quality through molecular breeding strategies.

## Supplementary information

Supplementary Table

Supplementary Figures 1-17

Supplementary Tables

Supplementary Tables

Supplementary Table

Supplementary Table

Supplementary Table

Supplementary Table

Supplementary Table

Supplementary Table

Supplementary Table

Supplementary Table

## Data Availability

The data generated herein to support the results of this study are presented in the paper and its Supplementary Information files. Moreover, the generated and analyzed datasets of this study are available from the corresponding authors upon request. The raw sequence data of *H. undatus* genome sequencing have been deposited in the SRA (Sequence Read Archive) data resource of the NCBI with the Bioproject ID PRJNA691451 (https://dataview.ncbi.nlm.nih.gov/object/PRJNA691451?reviewer=eoonj7im3bl6g7f4opdv05qv25) and on the figshare website (10.6084/m9.figshare.14456583). The transcriptome data of ‘GHB’ (*H. undatus*) and ‘GHH’ (*H. monacanthus*) was deposited under the Bioproject ID PRJNA704510 (https://dataview.ncbi.nlm.nih.gov/object/PRJNA704510?reviewer=ou0oqgevkctv5p68omqutsvsf9). The pitaya genome website can be viewed at http://www.pitayagenomic.com.
