# Supplementary material for: A chromosome-scale genome sequence of pitaya (Hylocereus undatus) provides novel insights into the genome evolution and regulation of betalain biosynthesis
Source: Hortic Res. 2021 Jul 6;8:164. doi: 10.1038/s41438-021-00612-0 (PMC8260669; doi:10.1038/s41438-021-00612-0)
Supplement: Supplementary file 2 — Supplementary Figures 1-17 [file 41438_2021_612_MOESM2_ESM.docx]

**Supplementary Figures**

| 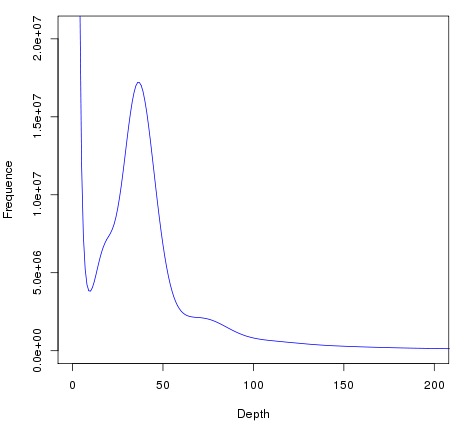 |
| --- |
| **Fig. 1 17-mer-based analysis to estimate the genome sizes of *H. undatus.*** |

| 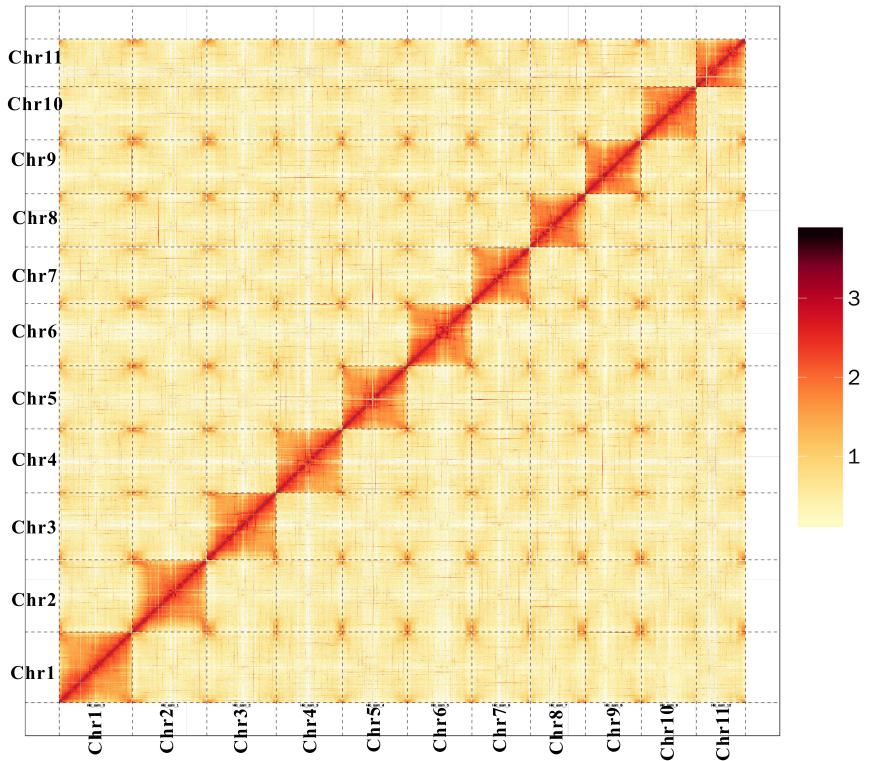 |
| --- |
| **Fig. 2 Chromosomal Hi-C contact map of the *H. undatus* genome.** The intensity of each pixel represents the count of Hi-C links between 500 kb windows on chromosomes on a logarithmic scale. The strongest and weakest contact are shown in red and yellow, respectively.   \| 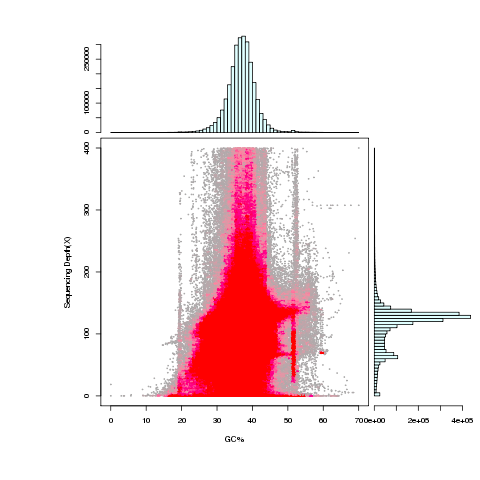 \| \| --- \| \| **Fig. 3 The distribution of GC content of *H. undatus* genome.** \| |

| 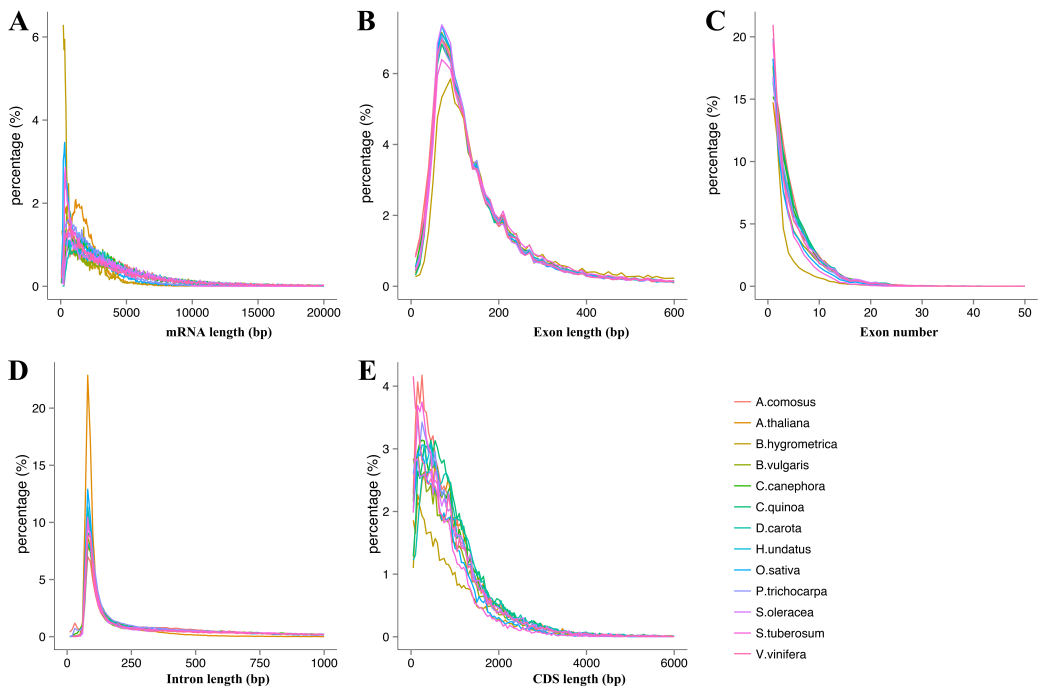 |
| --- |
| **Fig. 4 Comparison of mRNA length (A), CDS length (B), Exon length (C), Intron length (D), and Exon number per gene (E) between *H. undatus*, *A. thaliana*, *P. trichocarpa*, *O. sativa*, *A. comosus*, *C. quinoa*, *S. oleracea*, *B. vulgaris*, *V. vinifera*, *C. canephora*, *B. hygrometrica*, *S. tuberosum*, *D. carota.*** The x-axis represents length or number and the y-axis represents the density of genes. |

|  |
| --- |
| **Fig. 5 Gene family and genome evolution in *H. undatus*.** A, Syntenic depths in *H. undatus* vs. *S. oleracea* genome comparison. WGT, whole genome triplication event; WGD, whole-genome duplication event. B, 4dTv in *H. undatus* and two other eudicot plant species. |

| 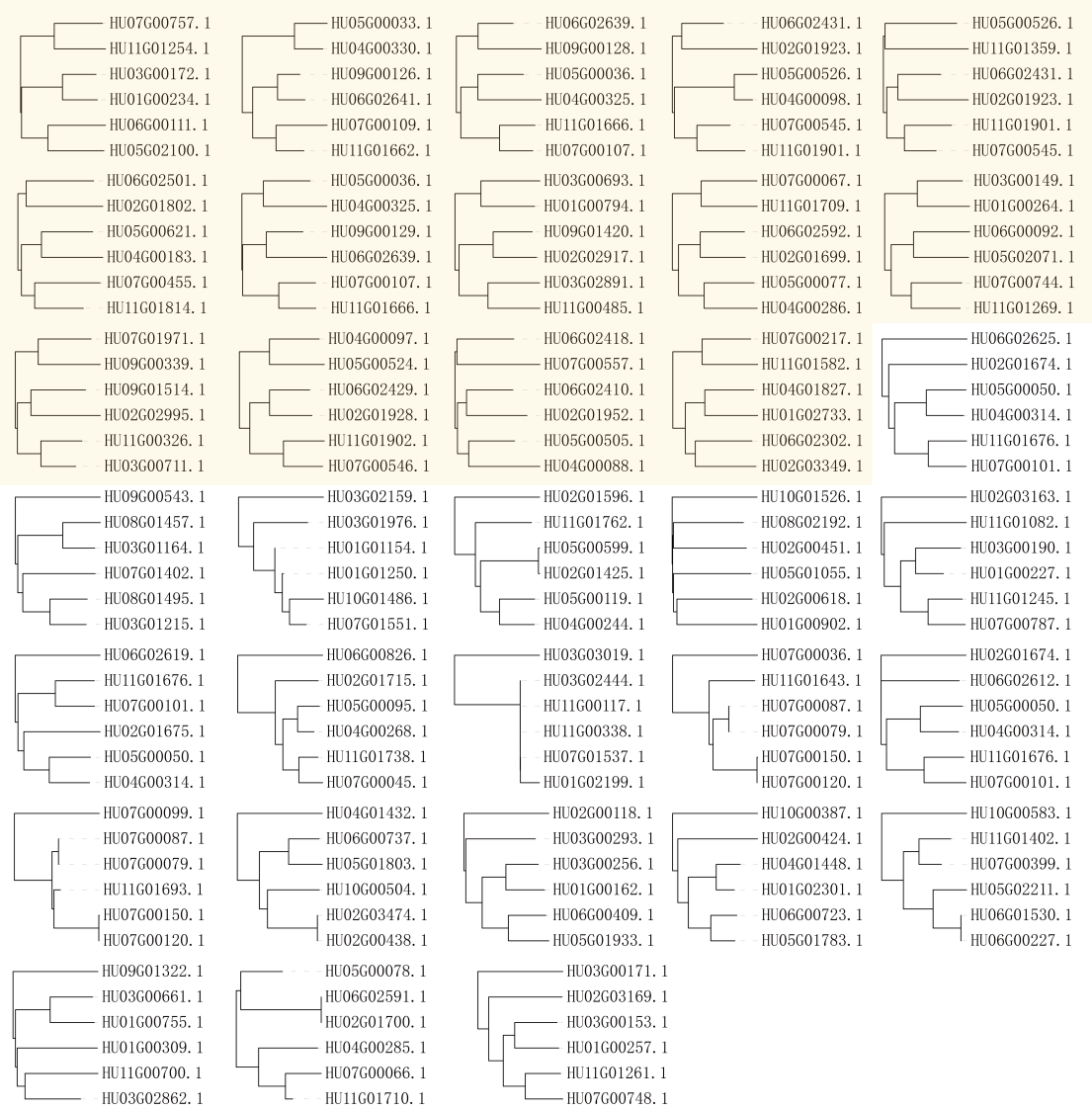 |
| --- |
| **Fig. 6 33 trees of 1:6 paralogous gene pairs. The light yellow background represents a recent WGD event after the WGT event.** |

**

**

**Fig. 7 Comparative genomic assessment.** **A,** Venn diagrams displays the gene family numbers shared among four Caryophyllales species. **B,** Gene distribution in different species.

| 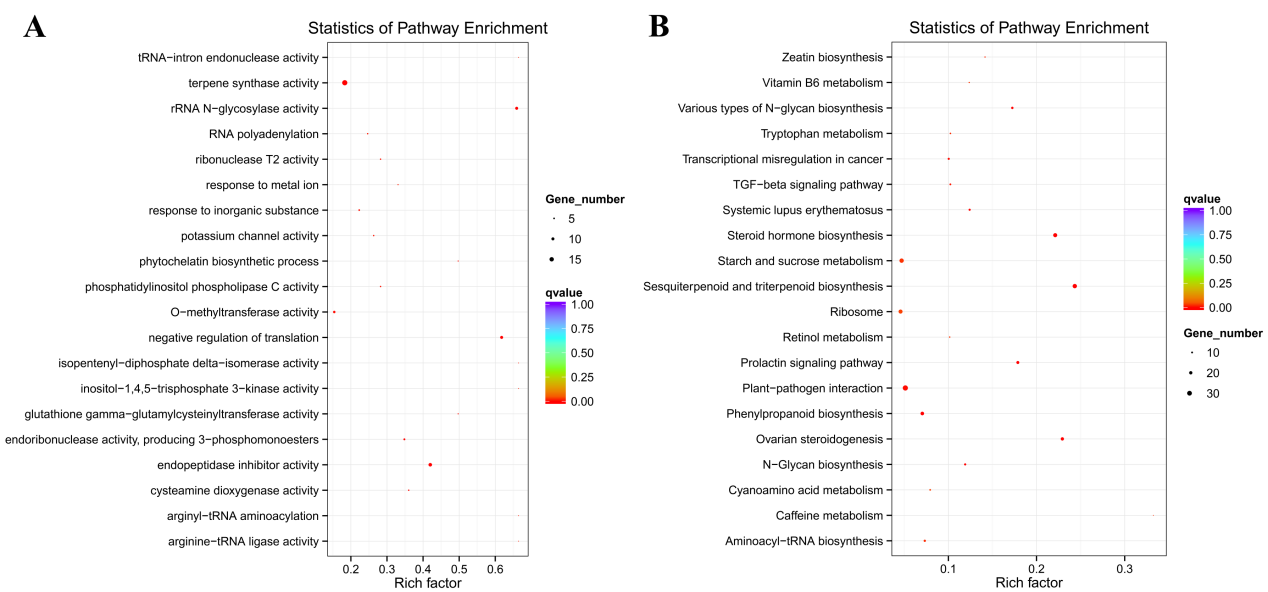 |
| --- |
| **Fig. 8 The GO (A) and KEGG (B) enrichment analysis of 517 unique gene families from *H. undatus* genome.** |


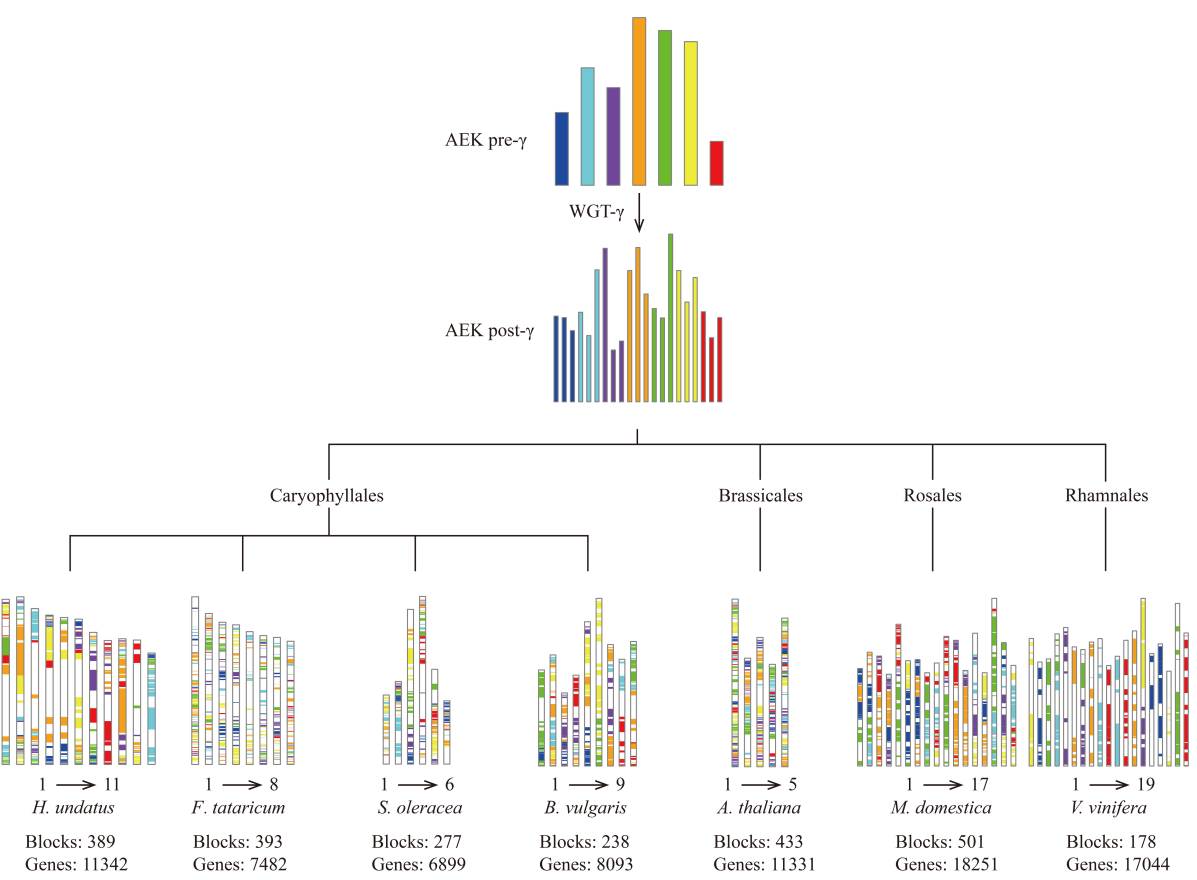


**Fig. 9 Modern chromosome derivation in *H. undatus* and six other grass species.**


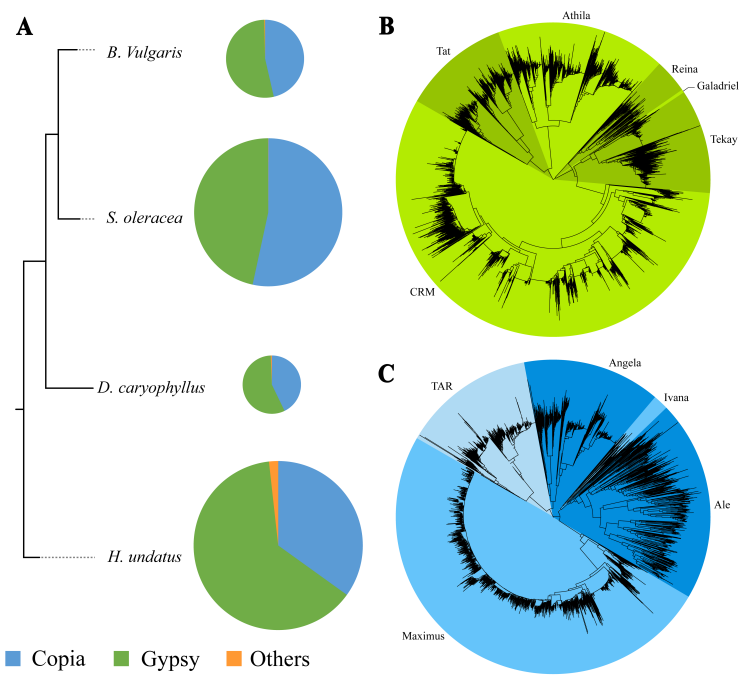


**Fig. 10 LTR-RTs contents and evolution of LTR-RTs super families. A,** LTR-RTs contents and proportions of different kinds of LTR-RTs in *H. undatus*, *S. oleracea*, *B. vulgaris* and *D. caryophyllus.* **B-C,** The neighbor-joining and unrooted phylogenetic trees were generated on the basis of Ty3/gypsy (**B**) and Ty1/copia (**C**) aligned sequences corresponding to the RT domains without premature termination codon. Major lineages are named and proportion of each is indicated.


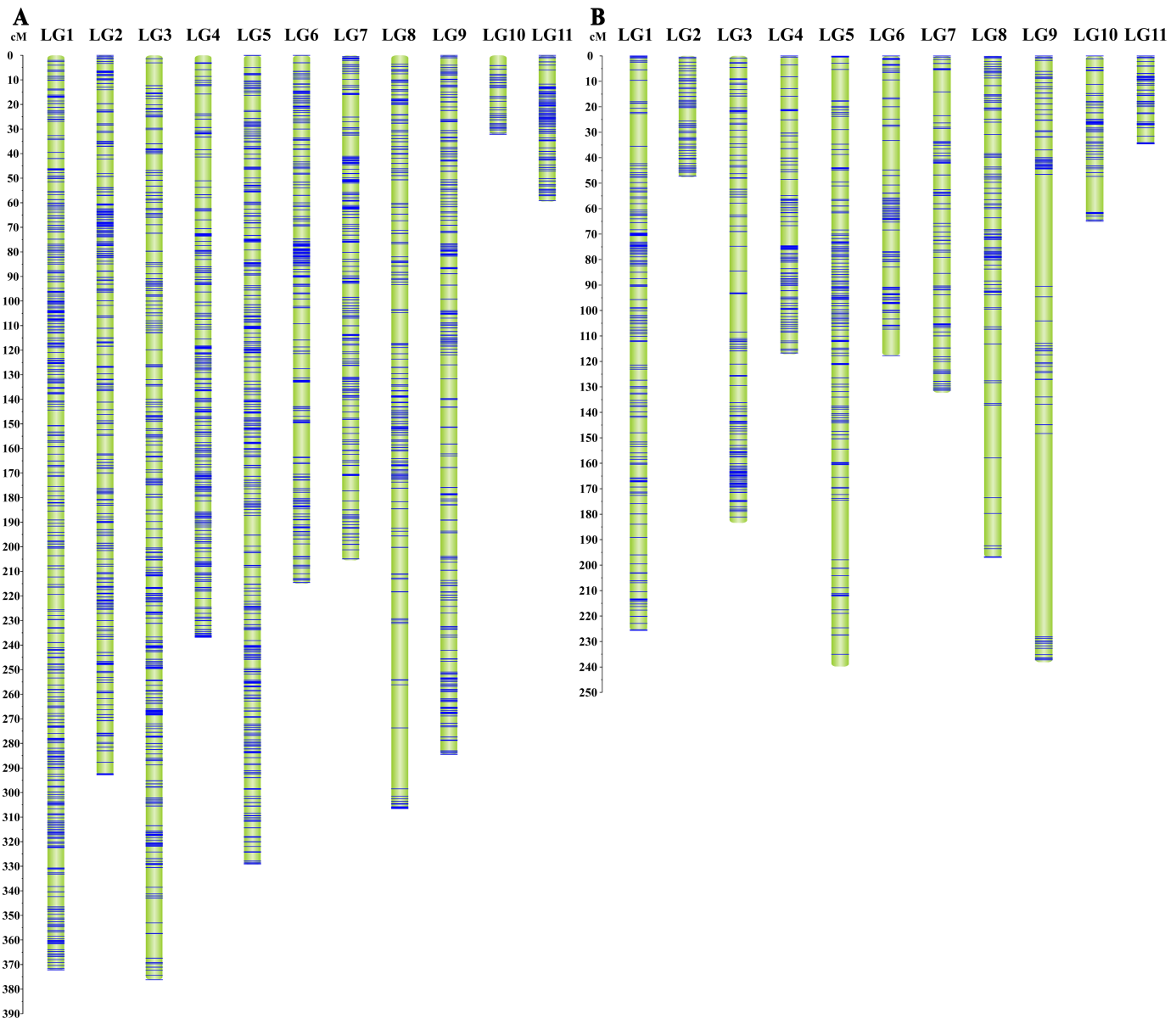


**Fig. 11** Genetic linkage groups of ‘GHB’ (**A**) and ‘Dahong’ pitayas (**B**) in 11 linkage groups. The scale at the left edge is genetic distance in centiMorgans (cM). LGs indicates linkage groups.


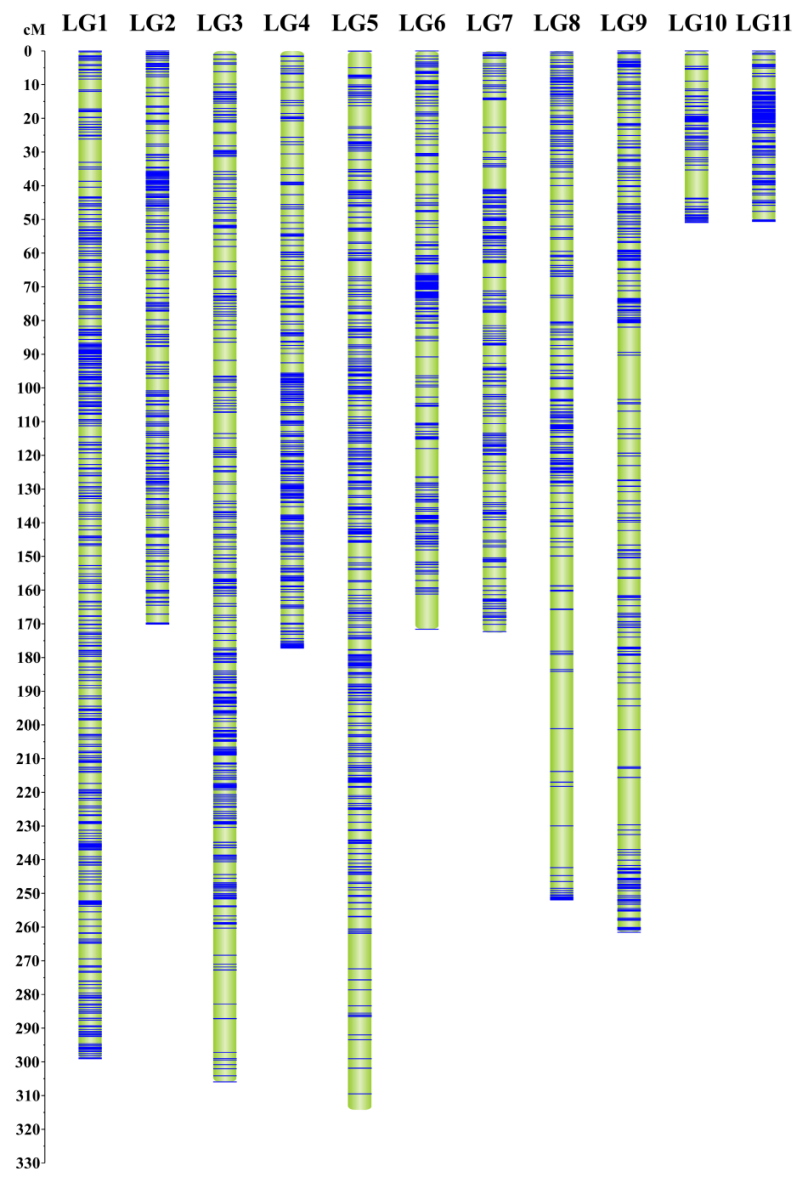


**Fig. 12** Integrated genetic linkage groups for F1 hybrid populations of ‘GHB’ × ‘Dahong’ pitayas and their parents based on 6,209 bin markers in 11 linkage groups. The scale at the left edge is genetic distance in cM. LGs indicates linkage groups.


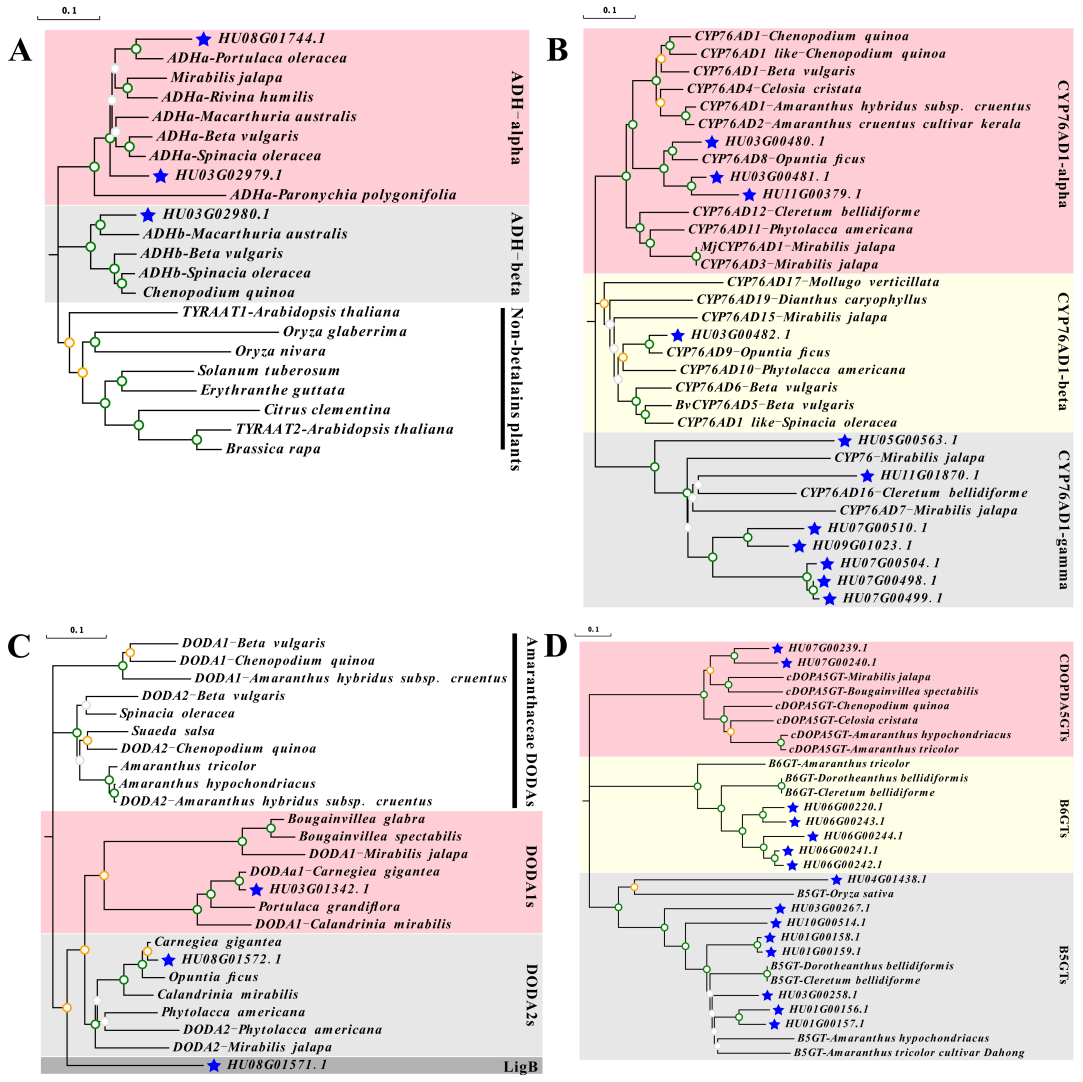


**Fig. 13 Phylogenetic trees of the ADH (A), CYP76AD1 (B), DODA (C) and GT (D) genes related to betalain biosynthetic pathway.** Maximum likelihood (ML) tree for each betalain biosynthetic gene was constructed using MEGA 7 with 1000 bootstrap replicates. Blue star indicates genes from *H. undatus* genome. Green, orange and grey circle in tree branch indicates the bootstrap value between 81-100, 60-80 and 0-59, respectively.


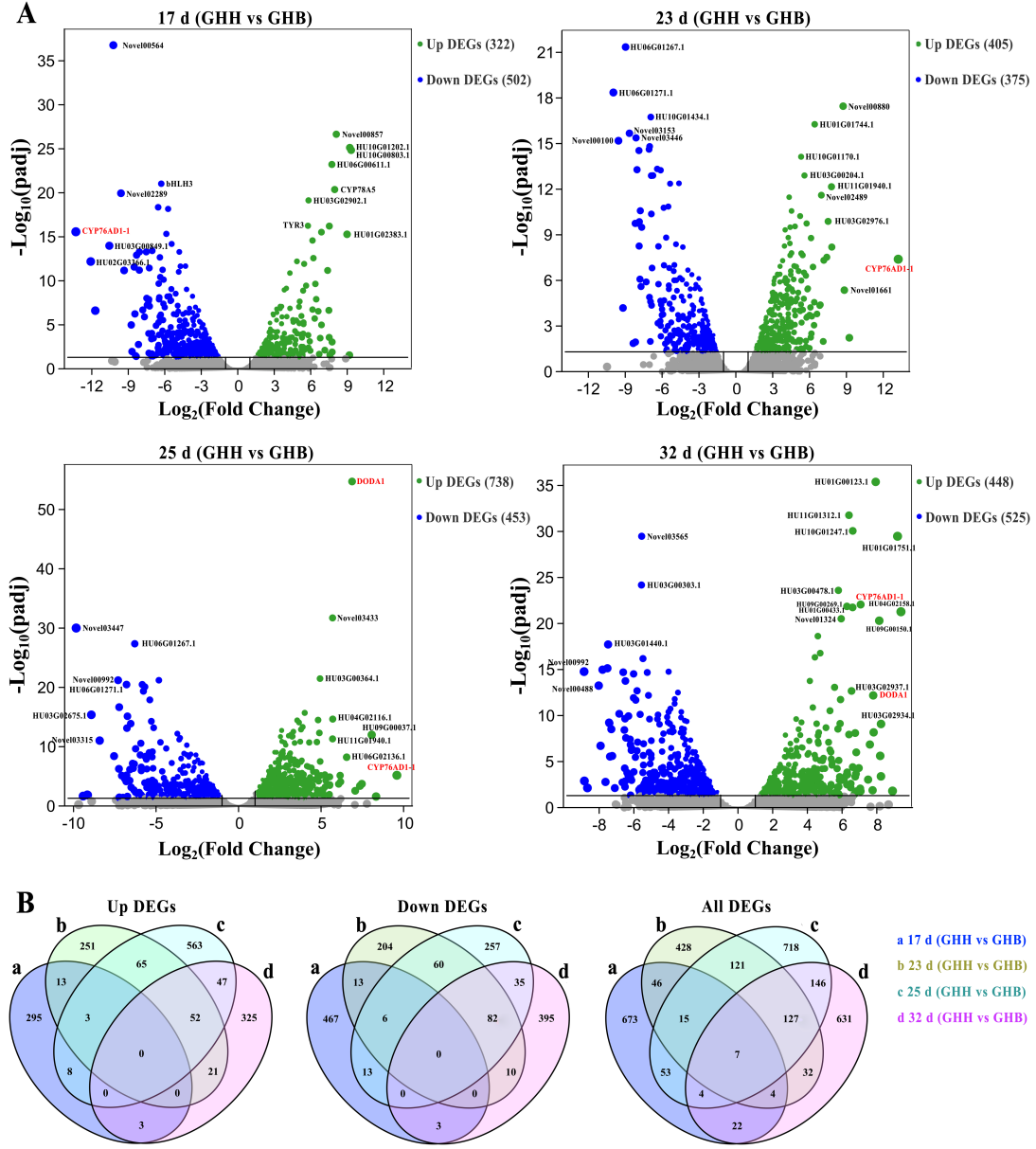


**Fig. 14 Differentially expressed genes (DEGs) of RNA-seq datasets. A,** Volcano plots of DEGs. Each dot in the Volcano plots of DEGs designates a gene. Abscissa is log_2_ (Fold Change) and ordinate is log_10_ (padj), both of them represented the logarithm of the multiple of gene expression difference between the two samples. The different color dots designate the genes expression differences. Amongst, the green and blue dots designate genes indicated the up- and down-regulated DEGs with the log_10_ (padj) > 0.05, respectively, while the grey dots designate the genes without significant expression difference with the log_10_ (padj) < 0.05. **B,** Venn diagrams show the comparison of the up-, down-regulated and all DEGs between two pitaya pulps of four stages.


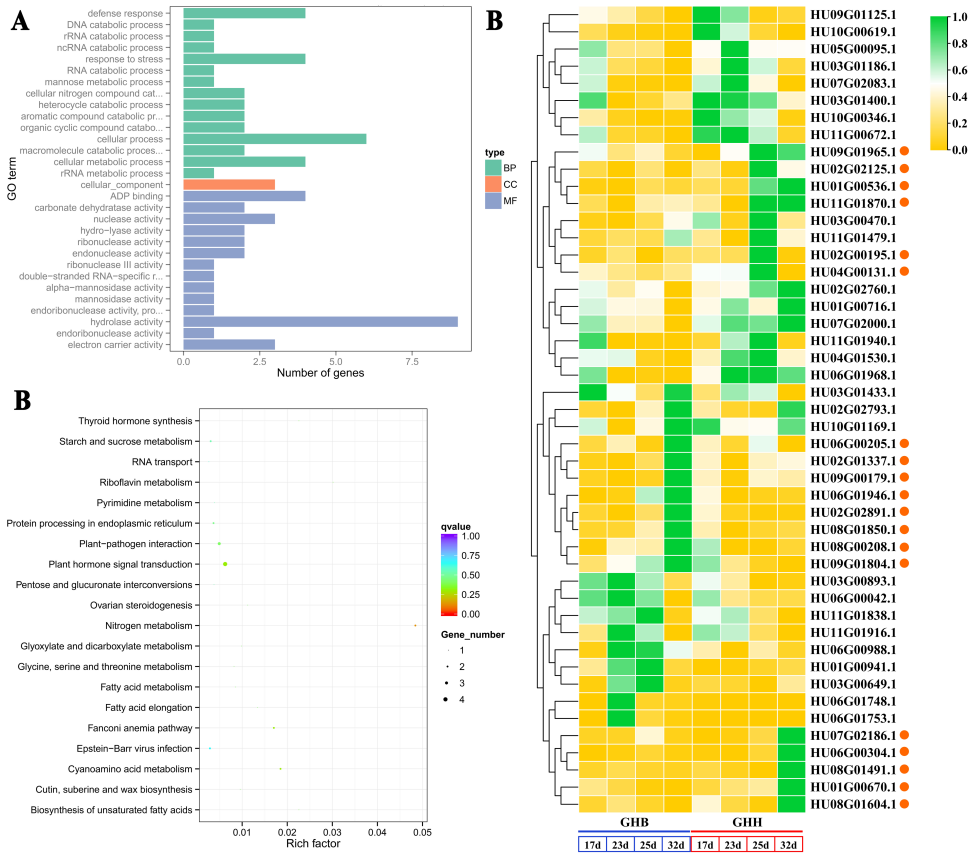


**Supplementary Fig. 15 Analyses of candidate genes involved in betalain biosynthesis from white and red pitaya pulp RNA-Seq datasets.** A-C, The GO (A), KEGG enrichment (B) and heatmap (C) of forty-seven candidate genes involved in betalain biosynthesis. Orange circles indicate candidate genes that only highly expressed in 32 d of ‘GHB’ or 25 d and 32 d of ‘GHH’ pitaya.

| 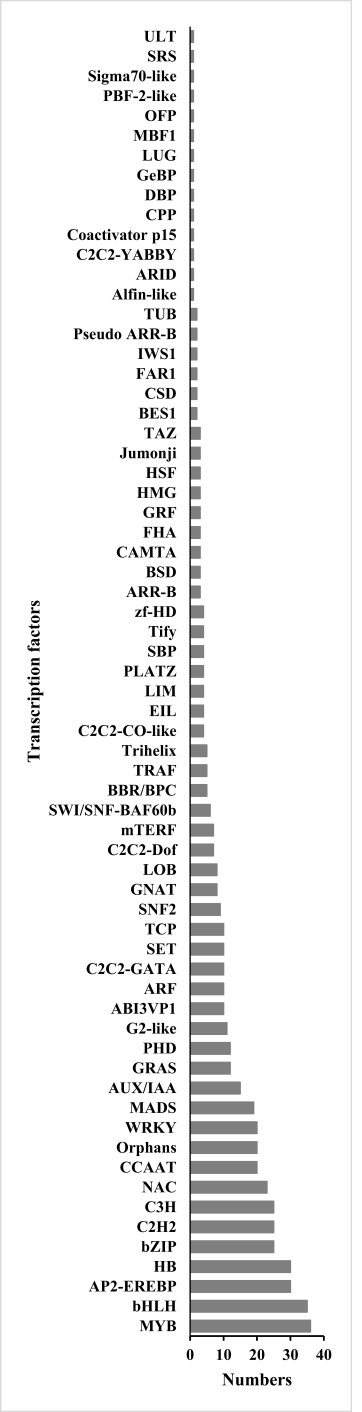 |
| --- |
| **Fig. 16 The statistics of 66 TF gene families from co-expression network.** |


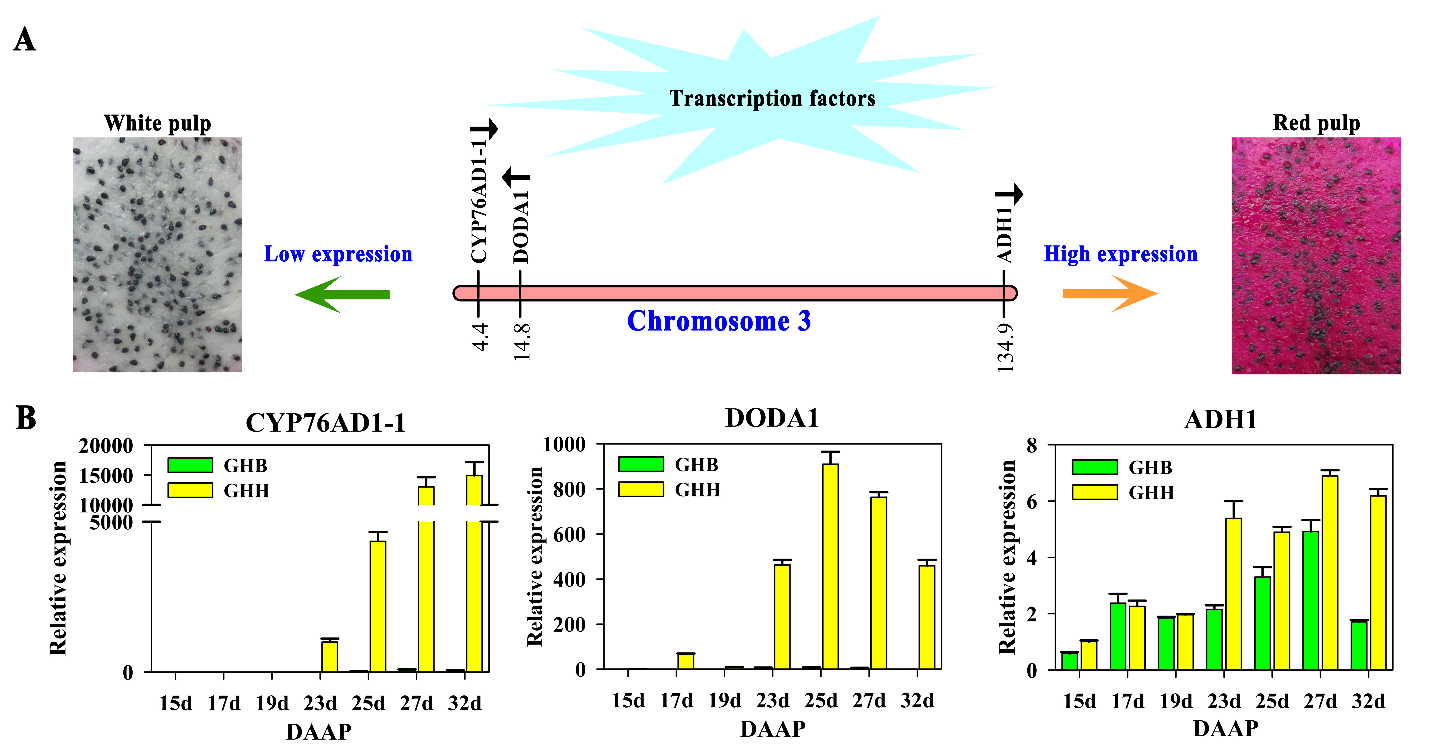


**Fig. 17 Schematic representation of the putative mechanism of two cultivars with different betalain levels in pitayas. A,** the location of key genes involved in betalain biosynthesis in chromosome 3. The unit is Mb, and the black arrows indicate transcriptional direction. **B,** The relative expression of key betalain biosynthetic genes in ‘GHB’ and ‘GHH’ pitayas.
