# Supplementary material for: A chromosome-scale genome sequence of pitaya (Hylocereus undatus) provides novel insights into the genome evolution and regulation of betalain biosynthesis
Source: Hortic Res. 2021 Jul 6;8:164. doi: 10.1038/s41438-021-00612-0 (PMC8260669; doi:10.1038/s41438-021-00612-0)
Supplement: Supplementary file 3 — Supplementary Tables [file 41438_2021_612_MOESM3_ESM.doc]

**Supplementary Tables**

| **Table 1 qRT-PCR primers used in this study** | |
| --- | --- |
| Primer name | Sequence (5'-3') |
| ADH1-F | TGATGATGGTGAGAAGACGA |
| ADH1-R | GTAGTCGGAGCGAGAGTAGG |
| CYP76AD1-1-F | GTAGTCGGAGCGAGAGTAGG |
| CYP76AD1-1-R | TCTTCCTAAAACTCCGCCAT |
| DODA1-F | TGTGCCTTCTGTATCTGCCG |
| DODA1-R | TGTCTTGAACCCTCCTGCTA |

| **Table 2 Survey statistic results of** H. undatus | | | | | | |
| --- | --- | --- | --- | --- | --- | --- |
| K-mer | K-mer number | K-mer depth | Genome size（Mb） | Revised genome size (Mb) | Heterozygous ratio (%) | Repeat (%) |
| 17 | 61,145,593,332 | 38 | 1609.09 | 1583.33 | 0.65 | 65.99 |

| **Table 3 Sequencing data used for *H. undatus* genome construction** | | | | |
| --- | --- | --- | --- | --- |
| Pair-end libraries | Insert size | Total data (Gb) | Read length (bp) | Sequence coverage (×) |
| Illumina reads | 250 bp | 75.92 | 150 | 48.05 |
|  | 350 bp | 78.42 |  | 49.63 |
|  | 450 bp | 76.3 |  | 48.29 |
|  | 2 kb | 75.59 |  | 47.84 |
|  | 5 kb | 76.08 |  | 48.15 |
|  | 10 kb | 41.52 |  | 26.28 |
| Pacbio reads | - | 178.91 | - | 113.23 |
| 10× Genomics | - | 237.98 | 150 | 150.62 |
| Total | - | 840.72 | - | 532.09 |

| **Table 4 Assemblies statistics for the** H. undatus **genome** | | | | |
| --- | --- | --- | --- | --- |
| Sample ID | Canu | | Hi-C | |
|  | Contig_len (Mb) | Contig_number | Scaffold_len (Mb) | Scaffold_number |
| Total | 1,412.88 | 126,556 | 1,386.95 | 870 |
| Max | 7.50 | - | 146.67 | - |
| Number ≥ 2 kb | - | 7,647 | - | 675 |
| N50 | 0.58 | 670 | 127.15 | 6 |

| **Table 5 Chromosomal Hi-C contact data mapped to the *H. undatus* genome** | | |
| --- | --- | --- |
| Chromosome | Length (Mb) | Percent (%) |
| 1 | 144.47 | 10.42 |
| 2 | 146.67 | 10.58 |
| 3 | 136.39 | 9.84 |
| 4 | 130.59 | 9.42 |
| 5 | 128.53 | 9.27 |
| 6 | 127.15 | 9.17 |
| 7 | 115.51 | 8.33 |
| 8 | 108.40 | 7.82 |
| 9 | 109.96 | 7.93 |
| 10 | 108.83 | 7.85 |
| 11 | 97.48 | 7.03 |
| Total | 1,353.96 | 97.67 |

| **Table 6 Genome completeness evaluated by Benchmarking Universal Single-Copy Orthologs (BUSCO)** | | | | |
| --- | --- | --- | --- | --- |
| Description | Assembly | | Annotation | |
|  | Number of genes | Percentage (%) | Number of genes | Percentage (%) |
| Complete BUSCOs | 1514 | 93.8 | 1477 | 91.5 |
| Complete and single-copy BUSCOs | 1437 | 89.0 | 1338 | 82.6 |
| Complete and duplicated BUSCOs | 77 | 4.8 | 144 | 8.9 |
| Fragmented BUSCOs | 42 | 2.6 | 66 | 4.1 |
| Missing BUSCOs | 58 | 3.6 | 71 | 4.4 |

| **Table 7 Assessment the gene coverage rate using eukaryotic genes using Core Eukaryotic Genes Mapping Approach (CEGMA)** | | |
| --- | --- | --- |
| Description | Fully mapped CEGMAs | Fully and partially mapped CEGMAs |
|  | Prots | % Completeness |
| Number of CEGs present in the assembly | 232 | 235 |
| Completeness of the genome (%) | 93.55 | 94.76 |

| **Table 8 Coverage statistics of *H*. *undatus* genome** | | |
| --- | --- | --- |
| Reads | Mapping rate (%) | 99.68 |
| Genome | Average sequencing depth | 125.2 |
|  | Coverage (%) | 95.20 |
|  | Coverage at least 4× (%) | 93.90 |
|  | Coverage at least 10× (%) | 92.98 |
|  | Coverage at least 20× (%) | 92.02 |

| **Table 9 The EST evaluation results of *H*. *undatus* genome** | | | | | | |
| --- | --- | --- | --- | --- | --- | --- |
| Dataset | Number | Total length (Mb) | With >90 % sequence in one scaffold | | With >50 % sequence in one scaffold | |
|  |  |  | Number | Percent (%) | Number | Percent (%) |
| >0 bp | 95,197 | 60.20 | 82,923 | 87.107 | 86,707 | 91.082 |
| >500 bp | 34,034 | 42.70 | 31,702 | 93.148 | 33,109 | 97.282 |
| >1 kb | 16,687 | 31.21 | 15,628 | 93.654 | 16,425 | 98.43 |
| >2 kb | 5,954 | 16.69 | 5,454 | 91.602 | 5,874 | 98.656 |

| **Table 10 Prediction of protein-coding genes in the *H*. *undatus* genome** | | | | | | | |
| --- | --- | --- | --- | --- | --- | --- | --- |
| Gene set | | Number | Average gene length (kb) | Average CDS length (bp) | Average exons per gene | Average exon length (bp) | Average intron length (bp) |
| *De novo* | Augustus | 40,767 | 3.40 | 944 | 4.11 | 230 | 815 |
|  | GlimmerHMM | 85,503 | 13.61 | 535 | 3.28 | 163 | 5887 |
|  | SNAP | 72,474 | 6.17 | 603 | 3.44 | 176 | 2360 |
|  | Geneid | 114,628 | 3.61 | 479 | 3 | 160 | 1614 |
|  | Genscan | 62,042 | 12.92 | 835 | 4.93 | 169 | 3151 |
| Homolog | *Chenopodium quinoa* | 60,588 | 2.50 | 912 | 2.79 | 327 | 920 |
|  | *Coffea canephora* | 45,822 | 2.63 | 953 | 3.02 | 316 | 865 |
|  | *Boea hygrometrica* | 51,141 | 2.33 | 1064 | 2.54 | 419 | 858 |
|  | *Populus trichocarpa* | 48,592 | 2.50 | 993 | 2.91 | 341 | 818 |
|  | *Ananas comosus* | 43,021 | 2.55 | 775 | 2.89 | 268 | 969 |
|  | *Oryza sativa* | 59,962 | 1.96 | 969 | 2.35 | 412 | 766 |
|  | *Solanum tuberosum* | 111,398 | 1.23 | 704 | 1.83 | 385 | 674 |
|  | *Vitis vinifera* | 42,609 | 2.78 | 937 | 3.14 | 298 | 890 |
|  | *Beta vulgaris* | 83,140 | 2.01 | 864 | 2.47 | 350 | 813 |
|  | *Spinacia oleracea* | 106,105 | 1.56 | 711 | 2.14 | 332 | 775 |
|  | *Arabidopsis thaliana* | 52,327 | 2.13 | 804 | 2.69 | 299 | 816 |
|  | *Daucus carota* | 39,162 | 3.72 | 1444 | 3.23 | 447 | 1064 |
| RNA-seq | Cufflinks | 36,433 | 6.12 | 1764 | 5.21 | 338 | 1068 |
|  | PASA | 44,573 | 5.20 | 1034 | 5.28 | 196 | 1001 |
| EVM | | 43,666 | 3.84 | 916 | 4.08 | 224 | 979 |
| PASA-update | | 43,533 | 3.87 | 919 | 4.09 | 225 | 985 |
| Final set | | 27,753 | 5.05 | 1159.29 | 5.06 | 229.22 | 957.78 |

| **Table 11 Functional annotation of the predicted genes for *H*. *undatus*** | | |
| --- | --- | --- |
| Database | Annotated number | Annotated percent (%) |
| NR | 26,313 | 94.9 |
| Swiss-Prot | 21,354 | 77.0 |
| KEGG | 20,016 | 72.2 |
| InterPro | 22,725 | 81.9 |
| Pfam | 21,352 | 77.0 |
| GO | 15,620 | 56.3 |
| Annotated | 26,371 | 95.1 |
| Total | 27,735 | - |

| **Table 13 Prediction of repetitive elements in the assembled *H*. *undatus*** | | |
| --- | --- | --- |
| Type | Repeat size (Mb) | % Genome |
| Trf | 140.06 | 10.3 |
| Repeatmasker | 835.14 | 61.4 |
| Proteinmask | 280.62 | 20.63 |
| Total | 896.31 | 65.9 |

| **Table 14 Summary of Repeat contents in *H*. *undatus*** | | | | | | | |
| --- | --- | --- | --- | --- | --- | --- | --- |
| Type | | | Length (Mb) | | | % Genome | |
| DNA | | | 113.21 | | | 8.32 | |
| LINE | | | 67.24 | | | 4.94 | |
| SINE | | | 4.20 | | | 0.31 | |
| LTR | | | 664.19 | | | 48.83 | |
| Satellite | | | 1.55 | | | 0.11 | |
| Simple repeat | | | 6.08 | | | 0.45 | |
| Unknown | | | 84.25 | | | 6.19 | |
| Total | | | 881.39 | | | 64.8 | |
| **Table 15 List of non-coding RNAs genes in the *H*. *undatus* genomes** | | | | | | | |
| Type | | Copy number | | Average length (bp) | Total length (kb) | | % Genome |
| miRNA | | 4989 | | 88 | 427.92 | | 0.0307 |
| tRNA | | 4857 | | 75 | 354.41 | | 0.0254 |
| rRNA | Total | 5909 | | 116 | 669.08 | | 0.0480 |
|  | 18S | 117 | | 300 | 34.33 | | 0.0025 |
|  | 28S | 68 | | 119 | 7.89 | | 0.0006 |
|  | 5.8S | 31 | | 108 | 3.28 | | 0.0002 |
|  | 5S | 5693 | | 112 | 623.58 | | 0.0448 |
| snRNA | Total | 3877 | | 115 | 434.72 | | 0.0312 |
|  | CD-box | 3146 | | 108 | 331.67 | | 0.0238 |
|  | HACA-box | 64 | | 134 | 8.40 | | 0.0006 |
|  | Splicing | 666 | | 145 | 94.53 | | 0.0068 |

| **Table 16 Summary of gene family clustering** | | | | | | |
| --- | --- | --- | --- | --- | --- | --- |
| Species | Total gene | Genes in families | Unclustered genes | Families | Unique families | Genes per family |
| *Hylocereus undatus* | 28,489 | 22,948 | 5,541 | 13,594 | 517 | 1.6881 |
| *Rhodiola crenulata* | 29,913 | 23,228 | 6,685 | 13,403 | 749 | 1.73304 |
| *Kalanchoe fedtschenkoi* | 30,964 | 24,994 | 5,970 | 14,327 | 576 | 1.74454 |
| *Arabidopsis thaliana* | 26,869 | 23,283 | 3,586 | 12,799 | 763 | 1.81913 |
| *Solanum lycopersicum* | 32,837 | 25,441 | 7,396 | 14,114 | 857 | 1.80254 |
| *Spinacia oleracea* | 25,178 | 19,674 | 5,504 | 13,877 | 577 | 1.41774 |
| *Beta vulgari* | 24,045 | 18,971 | 5,074 | 13,952 | 311 | 1.35973 |
| *Ananas comosus* | 26,442 | 21,168 | 5,274 | 12,187 | 880 | 1.73693 |
| *Ipomoea nil* | 50,980 | 48,405 | 2,575 | 14,381 | 1,296 | 3.3659 |
| *Phalaenopsis equestris* | 28,908 | 18,510 | 10,398 | 10,878 | 1,414 | 1.7016 |
| *Dianthus caryophyllus* | 43,280 | 37,615 | 5,665 | 12,759 | 1,690 | 2.94812 |

| **Table 19 Summary of intact LTRs with at least one protein-coding gene in *H. undatus*** | | | | | |
| --- | --- | --- | --- | --- | --- |
| Species | | *B. Vulgaris* | *D. caryophyllus* | *S. oleracea* | *H. undatus* |
| Copies | Ty1/*copia* | 2488 | 1445 | 7416 | 7611 |
|  | Ty3/*gypsy* | 2655 | 1761 | 5797 | 12220 |
|  | Others | 14 | 19 | 5 | 258 |
| Length (Mb) | Ty1/*copia* | 18.41 | 9.49 | 76.47 | 64.74 |
|  | Ty3/*gypsy* | 21.14 | 12.55 | 66.69 | 117.39 |
|  | Others | 0.12 | 0.13 | 0.05 | 3.24 |
| The proportion (%) of each super family | Ty1/*copia* | 48.25 | 44.81 | 56.11 | 37.89 |
|  | Ty3/*gypsy* | 51.48 | 54.60 | 43.86 | 60.83 |
|  | Others | 0.27 | 0.59 | 0.04 | 1.28 |

| **Table 20 The proportion (%) of lineages in Ty1/*copia* super family** | | | | | | |
| --- | --- | --- | --- | --- | --- | --- |
| Type | Bianca | Ivana | Maximus | Ale | TAR | Angela |
| *B. Vulgaris* | 5.71 | 3.36 | 14.07 | 57.39 | 9.28 | 10.19 |
| *D. caryophyllus* | 0.27 | 3.53 | 2.72 | 72.83 | 6.39 | 14.27 |
| *S. oleracea* | 0.52 | 0.93 | 6.01 | 14.47 | 5.41 | 72.65 |
| *H. undatus* | 0 | 1.54 | 49.97 | 20.63 | 13.8 | 14.07 |

| **Table 21 The proportion (%) of lineages in Ty3/*gypsy* super family** | | | | | | |
| --- | --- | --- | --- | --- | --- | --- |
| Type | Athila | CRM | Tekay | Galadriel | Reina | Tat |
| *B. Vulgaris* | 10.81 | 11.96 | 24.66 | 0.1 | 9.65 | 42.81 |
| *D. caryophyllus* | 12.04 | 8.95 | 19.14 | 0.31 | 3.4 | 56.17 |
| *S. oleracea* | 10.62 | 5.28 | 24.75 | 0.67 | 4.62 | 54.06 |
| *H. undatus* | 17.47 | 57.07 | 10.78 | 0.25 | 3.47 | 10.97 |
